# Autoantibodies are highly prevalent in non–SARS-CoV-2 respiratory infections and critical illness

**DOI:** 10.1172/jci.insight.163150

**Published:** 2023-02-08

**Authors:** Allan Feng, Emily Y. Yang, Andrew Reese Moore, Shaurya Dhingra, Sarah Esther Chang, Xihui Yin, Ruoxi Pi, Elisabeth K.M. Mack, Sara Völkel, Reinhard Geßner, Margrit Gündisch, Andreas Neubauer, Harald Renz, Sotirios Tsiodras, Paraskevi C. Fragkou, Adijat A. Asuni, Joseph E. Levitt, Jennifer G. Wilson, Michelle Leong, Jennifer H. Lumb, Rong Mao, Kassandra Pinedo, Jonasel Roque, Christopher M. Richards, Mikayla Stabile, Gayathri Swaminathan, Maria L. Salagianni, Vasiliki Triantafyllia, Wilhelm Bertrams, Catherine A. Blish, Jan E. Carette, Jennifer Frankovich, Eric Meffre, Kari Christine Nadeau, Upinder Singh, Taia T. Wang, Eline T. Luning Prak, Susanne Herold, Evangelos Andreakos, Bernd Schmeck, Chrysanthi Skevaki, Angela J. Rogers, Paul J. Utz

**Affiliations:** 1 Department of Medicine, Division of Immunology and Rheumatology,; 2 Institute for Immunity, Transplantation and Infection,; 3 Department of Medicine, Division of Pulmonary, Allergy and Critical Care Medicine, and; 4 Department of Medicine, Division of Infectious Diseases, Stanford University School of Medicine, Stanford, California, USA.; 5 Department of Hematology, Oncology, Immunology, Philipps University Marburg, Marburg, Germany.; 6 Institute of Laboratory Medicine, Universities of Giessen and Marburg Lung Center (UGMLC), Philipps University Marburg, German Center for Lung Research (DZL), Marburg, Germany.; 7 4th Department of Internal Medicine, Medical School, National and Kapodistrian University of Athens, Attikon University Hospital, Athens, Greece.; 8 European Society of Clinical Microbiology and Infectious Diseases (ESCMID), Study Group for Respiratory Viruses (ESGREV), Basel, Switzerland.; 9 Department of Emergency Medicine and; 10 Department of Microbiology and Immunology, Stanford University School of Medicine, Stanford, California, USA.; 11 Laboratory of Immunobiology, Center for Clinical, Experimental Surgery and Translational Research, Biomedical Research Foundation of the Academy of Athens, Athens, Greece.; 12 Institute for Lung Research, UGMLC, Philipps University Marburg, Marburg, Germany.; 13 Chan Zuckerberg Biohub, San Francisco, California, USA.; 14 Department of Pediatrics, Division of Allergy, Immunology, Rheumatology, Stanford University School of Medicine, Stanford, California, USA.; 15 Department of Immunobiology, Yale University, New Haven, Connecticut, USA.; 16 Department of Medicine, Sean N. Parker Center for Allergy and Asthma Research, Stanford University School of Medicine, Stanford, California, USA.; 17 Department of Pathology and Laboratory Medicine and; 18 Institute for Immunology, Perelman School of Medicine, University of Pennsylvania, Philadelphia, Pennsylvania, USA.; 19 Department of Internal Medicine V, Infectious Diseases and Infection Control, UKGM, Justus Liebig University, and Institute for Lung Health (ILH), Giessen, Germany.; 20 DZL and UGMLC, Giessen, Germany.; 21 Department of Medicine, Pulmonary and Critical Care Medicine, University Medical Center Marburg, Marburg, Germany.; 22 DZL, German Center for Infection Research (DZIF), Center for Synthetic Microbiology (SYNMIKRO), Philipps University of Marburg, Marburg, Germany.

**Keywords:** Autoimmunity, Infectious disease, Adaptive immunity, Bacterial infections, Influenza

## Abstract

The widespread presence of autoantibodies in acute infection with SARS-CoV-2 is increasingly recognized, but the prevalence of autoantibodies in non–SARS-CoV-2 infections and critical illness has not yet been reported. We profiled IgG autoantibodies in 267 patients from 5 independent cohorts with non–SARS-CoV-2 viral, bacterial, and noninfectious critical illness. Serum samples were screened using Luminex arrays that included 58 cytokines and 55 autoantigens, many of which are associated with connective tissue diseases (CTDs). Samples positive for anti-cytokine antibodies were tested for receptor blocking activity using cell-based functional assays. Anti-cytokine antibodies were identified in > 50% of patients across all 5 acutely ill cohorts. In critically ill patients, anti-cytokine antibodies were far more common in infected versus uninfected patients. In cell-based functional assays, 11 of 39 samples positive for select anti-cytokine antibodies displayed receptor blocking activity against surface receptors for Type I IFN, GM-CSF, and IL-6. Autoantibodies against CTD-associated autoantigens were also commonly observed, including newly detected antibodies that emerged in longitudinal samples. These findings demonstrate that anti-cytokine and autoantibodies are common across different viral and nonviral infections and range in severity of illness.

## Graphical abstract

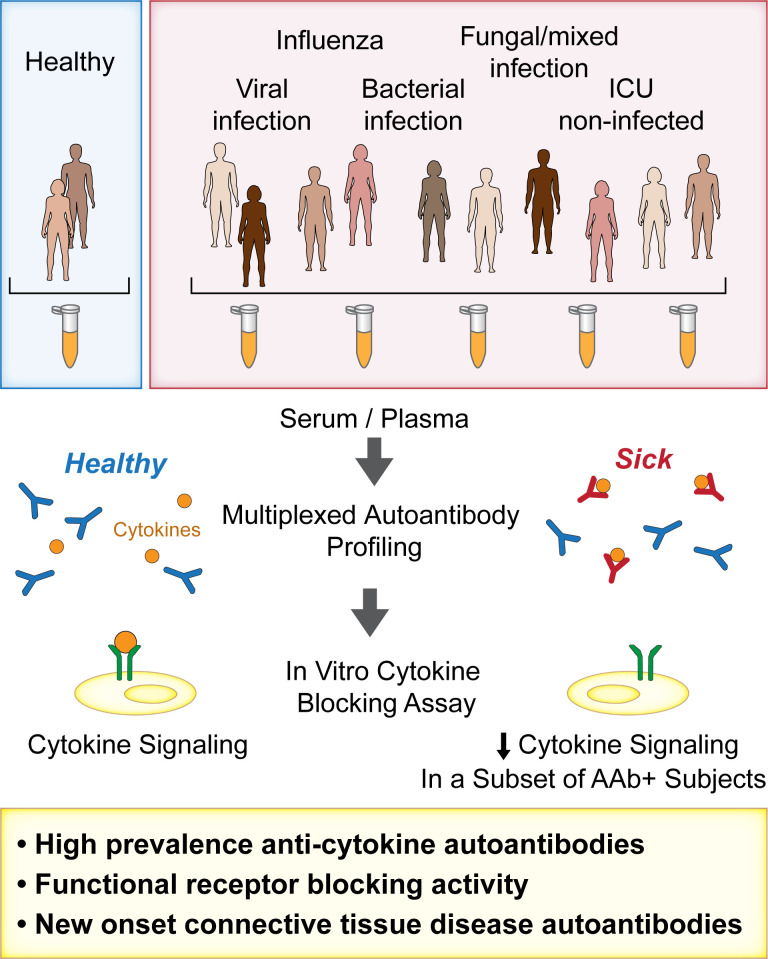

## Introduction

A pathogenic role for autoantibody formation in patients with SARS-CoV-2 is increasingly recognized (1–4). More than 60% of hospitalized patients with COVID-19 have 1 or more antibodies that recognize cytokines (anti-cytokine antibodies [ACA]) (3, 5, 6). We recently described the presence of IgG autoantibodies in most patients with COVID-19, including ACA and antibodies to intracellular antigens associated with rare connective tissue diseases (CTDs), such as systemic sclerosis (SSc), myositis, and overlap syndromes (5). Serum autoantibodies have been proposed to cause or contribute to clinical manifestations, such as more severe respiratory failure, vasculitis, and thrombosis in COVID-19 (1–4). In addition to their clear role in COVID-19, ACA are known to be associated with several lung diseases, such as disseminated atypical mycobacterial infections (AMI; associated with anti–IFN-γ; ref. 7) and pulmonary alveolar proteinosis (PAP; associated with anti–GM-CSF; ref. 8).

Whether widespread auto-antibody formation and loss of tolerance occurs in acute infections caused by other pathogens is unknown. We tested this hypothesis by screening for antibodies against cytokines and other autoantigens in blood samples from 5 cohorts without SARS-CoV-2, across a range of infection status and severity of illness. Among ICU patients, we found that those who had infections had a higher prevalence of ACA than patients who were thought to be uninfected. This finding was observed in all 5 patient cohorts. In addition, autoantibodies and ACAs with blocking activity were found in patients who were clinically phenotyped with only bacterial infection. CTD autoantibodies were also common, including some that emerged over the course of illness. Taken together, this study suggests that autoimmunity is linked not only to SARS-CoV-2, but also to other classes of pathogenic infections.

## Results

### Samples.

Blood samples in this study came from 267 patients recruited from 4 centers over 5 years, making up 5 non–SARS-CoV-2 cohorts of respiratory illness (Table 1). These cohorts include patients with a range of illness, with the majority presenting with viral, bacterial, and noninfectious critical illness (Supplemental Tables 1–5; supplemental material available online with this article; https://doi.org/10.1172/jci.insight.163150DS1). The cohorts included (a) patients admitted to the Stanford Hospital ICU with at least 1 acute respiratory distress syndrome (ARDS) risk factor (*n* = 167; Supplemental Table 1); (b) patients from Philipps University Marburg who were hospitalized for COVID-19 symptoms but tested negative by PCR (*n* = 19; Supplemental Table 2); (c) patients from the Phillips University Marburg who were hospitalized with Influenza A and pneumonia (*n* = 25; Supplemental Table 3); (d) patients with ARDS from the University of Giessen (*n* = 17; Supplemental Table 4); and (e) patients with acute influenza admitted to or treated as outpatients at the Sotiria Thoracic Diseases Hospital of Athens and the Attikon University Hospital (*n* = 40; Supplemental Table 5; ref. 9). Positive control samples were derived from patients with autoimmune diseases with known reactivity patterns and blocking activity. Healthy controls (HC) (*n* = 30) were obtained from Stanford Blood Bank and Stanford Hospital and Clinics.

### ACA are highly prevalent in all cohorts of acute illness.

**Figure 1:**
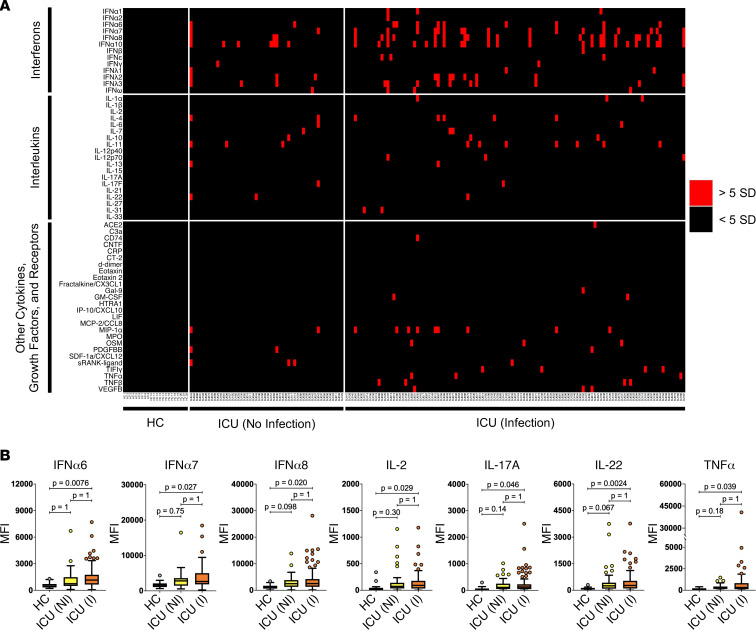
High prevalence of ACA in hospitalized ICU patients. (**A**) Heatmap representing serum IgG ACA discovered using a 58-plex array of cytokines, chemokines, growth factors, and receptors. Stanford ICU patients who were infected with viruses, bacteria, fungi, or a combination of pathogens (*n* = 115), Stanford ICU patients with no evidence for infection (*n* = 52), and HC (*n* = 22) were analyzed for ACA. Cytokines are grouped on the *y* axis by category (IFNs, ILs, and other cytokines/growth factors/receptors). Colors indicate ACA whose MFI measurements are > 5 SD (red) or < 5 SD (black) above the average MFI for HC. MFIs < 3,000 were excluded. (**B**) Tukey box plots comparing MFI data from HC and Stanford ICU patients for the 7 antigens for which statistically significant differences (*P* < 0.05) were determined between patient groups using 2-tailed Wilcoxon rank-sum tests with Bonferroni correction. The middle line represents the median, while the lower and upper hinges correspond to the first and third quartiles. The upper whisker extends from the hinge to 1.5 times the interquartile range (IQR) above the 75th percentile MFI value, and the lower whisker extends from the hinge to 1.5 times the IQR below the 25th percentile MFI value.

**Figure 2:**
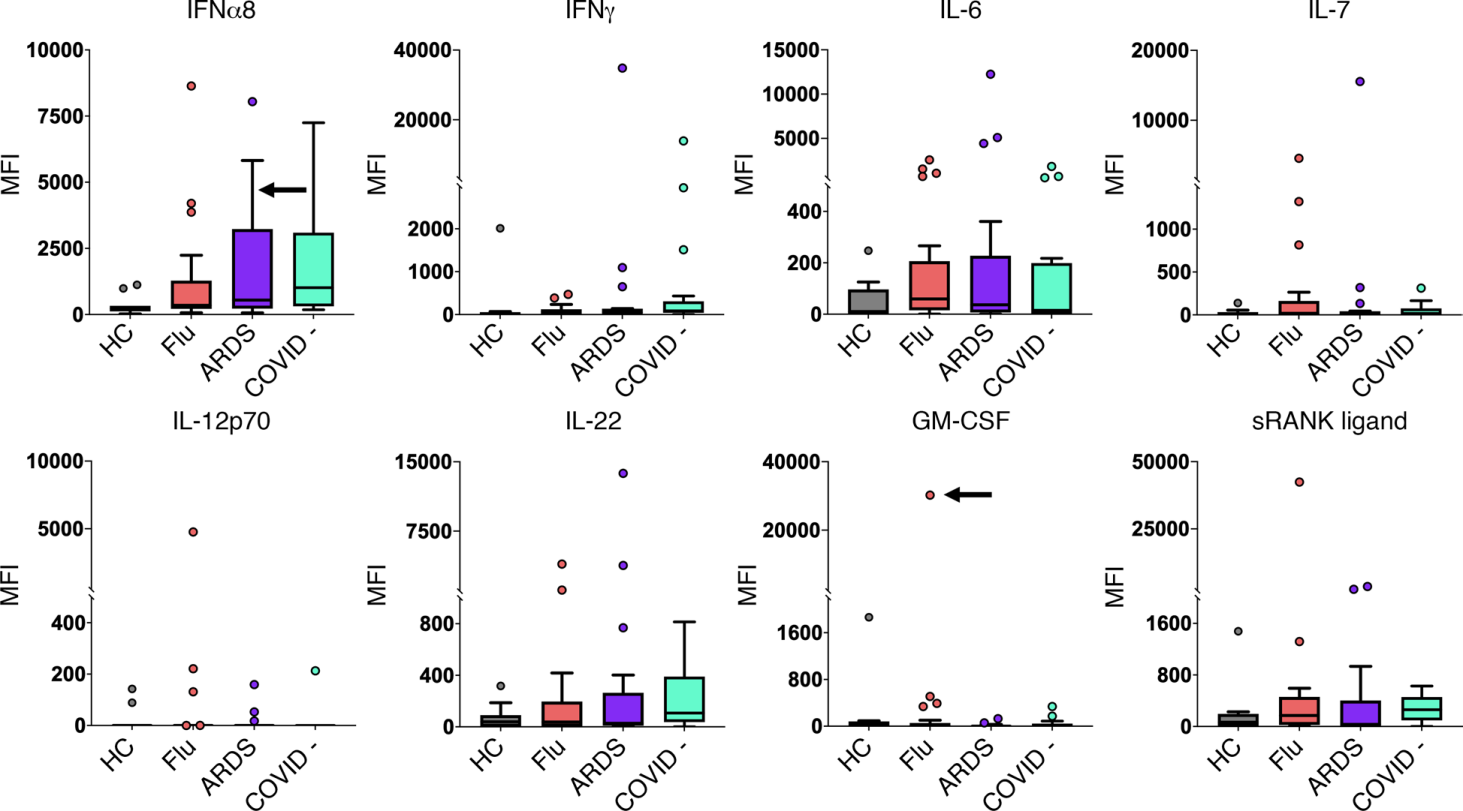
IgG anti-cytokine autoantibodies in serum from patients with ARDS or patients acutely infected with influenza virus. Tukey box plots comparing MFI data for 8 cytokines in patients with influenza (*n* = 25) and patients with ARDS (*n* = 17), both collected prior to the COVID-19 pandemic; patients with ARDS who were COVID-19^–^ (*n* = 19); and HC (*n* = 11). One COVID-19 PCR-negative patient from the Marburg cohort had high levels of antibodies targeting SARS-CoV-2 proteins from our viral array and was excluded from this figure and other analyses (Supplemental Figure 6). The middle line represents the median, while the lower and upper hinges correspond to the first and third quartiles. The upper whisker extends from the hinge to 1.5 times the IQR above the 75th percentile MFI value, and the lower whisker extends from the hinge to 1.5 times the IQR below the 25th percentile MFI value. Black arrows indicate a serum sample with receptor-blocking activity (see Figure 4). Individual MFI values 1.5 times the IQR above the 75th percentile or 1.5 times the IQR below the 25th percentile are displayed as dots. MFI is shown on the *y* axis, which is hatched to reflect outlier samples with very high MFI. Cohorts are shown on the *x* axis.

We used a custom 58-plex cytokine array (Supplemental Table 6) to screen samples for ACA and identify autoantibody-positive samples (median fluorescence intensity [MFI] > 5 SDs above mean HC MFI and above a 3,000 static cutoff). As shown in Table 1, we detected ACA in every cohort (range 40%–59% of individuals with at least 1 autoantibody across the 5 cohorts). In the Stanford ICU cohort, 51% of individuals were positive for at least 1 autoantibody versus 0% in 22 HC (*P* < 0.01) (Figure 1). Findings in the other 4 viral and ARDS cohorts were similar when compared with 11 HC (0% ACA) (Supplemental Figure 1). Striking reactivities were observed particularly for IFN-α8, IFN-γ, IL-6, IL-7, IL-12p70, and IL-22, while serum from 1 patient with influenza showed high MFI levels for both GM-CSF and soluble RANK ligand (Figure 2).

To ensure robustness of these findings, we assessed significance by comparing absolute MFI in the Stanford ICU cases and HC using 2-sided Wilcoxon rank-sum tests with Bonferroni correction (Supplemental Figure 2) and separately using the Significance analysis of microarrays (SAM) algorithm (Supplemental Figure 3), and we confirmed significant differences between cases and controls for 9 and 15 ACA, respectively. A subset of the statistically significant cytokines (e.g., IFN-α, IFN-ε, IL-22, and TNF-α) have previously been identified as common autoantibody targets in hospitalized patients with COVID-19 (5).

### ACA are more common in infected versus uninfected critically ill adults.

The Stanford ICU cohort enrolled critically ill patients admitted with at least 1 ARDS risk factor and included 69% infected and 31% uninfected patients by consensus physician phenotyping (Figure 1A and Supplemental Table 1). Using 2-sided Wilcoxon rank-sum tests with Bonferroni correction, we tested the hypothesis that ACA were specifically associated with infection. We found that 69 of 115 (60%) serum samples from infected patients were positive for at least 1 ACA, versus 17 of 52 (33%) positive for ACA in uninfected patients (OR, 3.1; 95% CI, 1.5–6.6; *P* = 0.001) (Supplemental Table 7). Logistic regression analysis adjusting for baseline characteristics (age, sex, and race) as well as a fully adjusted model (baseline characteristics, shock, and APACHE II score) both confirmed infection status as a significant predictor of presence of ACA (*P* < 0.001). Seven of the 9 statistically significant antigens, including IFN-α, IL-2, IL-17A, IL-22, and TNF-α, between the overall Stanford ICU cohort and HC group (Supplemental Figure 2) were statistically significant when comparing the infected ICU and HC groups (Figure 1B).

We next compared the presence of ACA in the Stanford ICU cohort based on the class of pathogen. Of 83 patients with only bacterial infections, 50 patients (60%) had ACA, compared with 33% of patients with no infections (*n* = 52; OR, 3.1; 95% CI, 1.4–6.9; *P* = 0.003) (Supplemental Figure 4). Within the mixed/fungal group, the subgroup of patients with both viral and bacterial infections had the highest rate of ACA (*n* = 7 of 10; OR, 4.7; 95% CI, 0.9–31.5; *P* = 0.037) (Supplemental Figure 4), while 3 of 3 (100%) patients with only fungal infections had at least 1 ACA. No significant differences were observed when comparing patients with only viral infections and uninfected patients (7 of 16 [44%]; *P* = 0.55).

We determined whether any of the antibodies to the 7 cytokines identified in Figure 1B were uniquely identified in a particular class of pathogens. Antibodies specific for IFN-α7 and IL-22 were significantly more prevalent in patients with only bacterial infections and patients with mixed bacterial/fungal infections, and antibodies against IFN-α10, IL-17A, and TNF-α were more common in patients with mixed bacterial/fungal infections (Supplemental Figure 4). No significant reactivities were found in patients with viral infections for the 7 cytokines, likely due to a small sample size. Taken together, these data suggest that the presence of serum ACA is associated not just with SARS-CoV-2, but with a broad spectrum of pathogens that cause ICU-associated infections.

### Autoantibodies that recognize common autoantigens, particularly those associated with CTDs, are highly prevalent in critically ill patients but do not differ by infection status.

We identified 102 of 167 (61%) Stanford ICU patient serum samples that were positive for common autoantibodies (termed CTD-AAb since a majority of the antigens are CTD targets) recognizing at least 1 of 55 common autoantigens in our array (Supplemental Table 8), particularly those associated with myositis (Supplemental Figure 5). None of the 14 HC samples were positive for any of the 55 CTD-AAb. Antibodies recognized tRNA synthetases PL-7 and EJ in 18 (11%) and 14 (8%) samples of 167 total, respectively, while anti-MDA5 antibodies were identified in 13 individuals. TPO was also commonly recognized by antibodies in 18 (11%) samples. SAM analysis revealed that 10 of the 55 antigens had significantly higher MFI levels in the ICU cohort compared with HC (Supplemental Figure 3B). These findings were similar in the 4 additional cohorts, with a CTD-AAb prevalence of 50% across all cohorts (Supplemental Figure 5).

There was no difference in CTD-AAb prevalence when comparing infected and uninfected Stanford ICU patients, with 59% of patients with infection having a CTD-AAb compared with 65% of uninfected patients (*P* = 0.5) (Supplemental Table 7). Autoantibodies were more common in older patients (66% age ≥ 60 versus 50% age < 60; *P* = 0.04). Logistic regression analysis evaluating baseline characteristics showed that higher age was associated with CTD-AAb status (*P* = 0.04). A fully adjusted logistic regression model including baseline characteristics as well as shock and APACHE II score showed that higher age (*P* = 0.006) and lower APACHE II score (*P* = 0.05) were significant predictors of CTD-AAb development. Infection was not associated with presence of autoantibodies in these adjusted models. Thus, while CTD-AAbs are markedly higher in all 5 cohorts than HC, they are not as clearly associated with infection as ACA.

### Longitudinal profiling of CTD-AAbs identifies newly detectable autoantibodies that were not present in the baseline sample.

**Figure 3:**
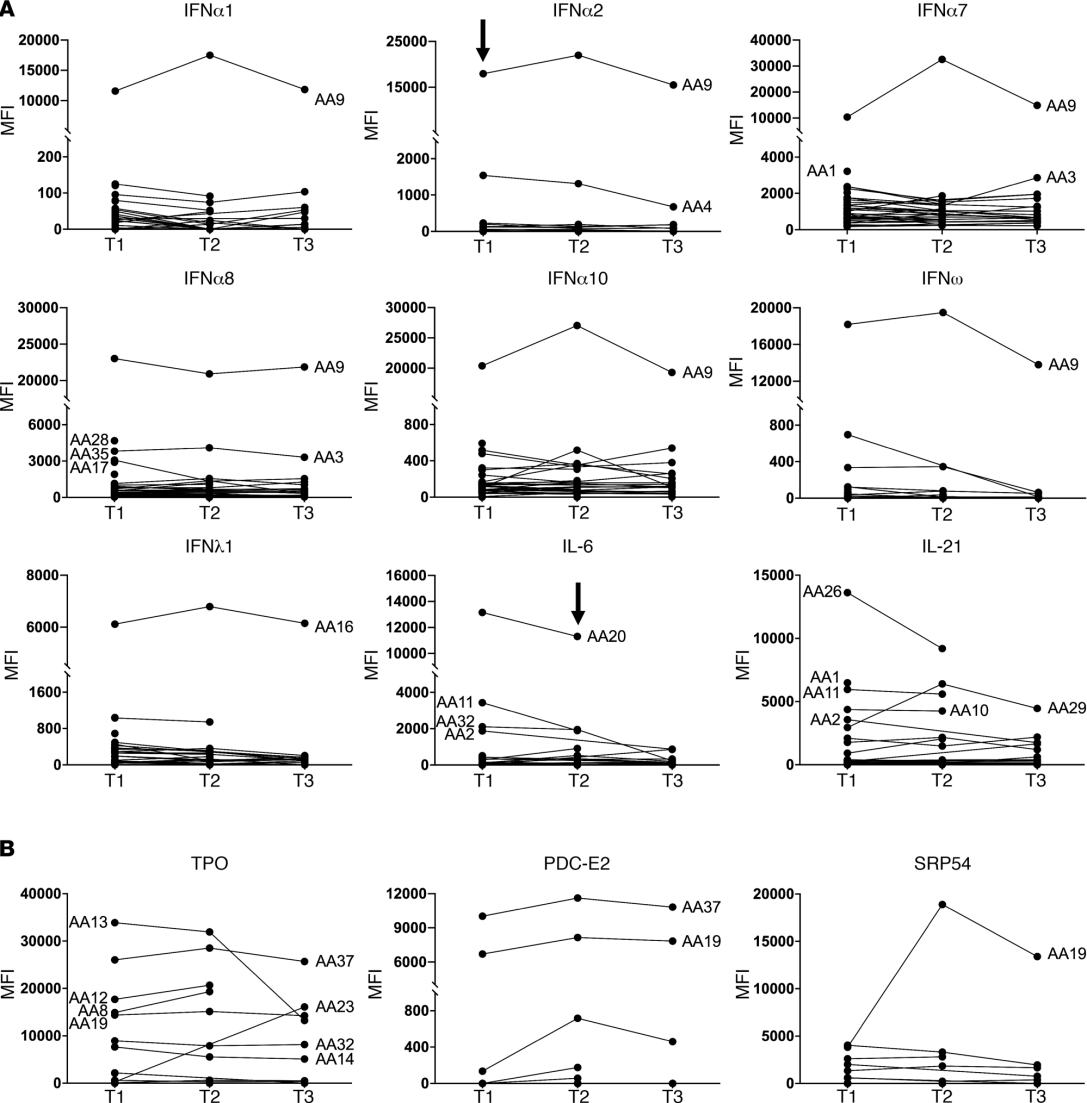
Newly detectable autoantibodies in acutely infected patients with influenza. (**A**) Longitudinal measurements of specific ACA over time in acutely infected patients (*n* = 40). Serum was collected at 3 time points for 18 influenza individuals, at 2 time points for 13 influenza individuals, and at only the first time point for 9 individuals. The first time point (T1) is from the day that the patient was admitted to the hospital and diagnosed with influenza. T2 and T3 refer to approximately 1 week and 1 month, respectively, following hospital admission. Black arrows indicate a serum sample with receptor blocking activity (see Figure 4). (**B**) Newly detectable IgG autoantibodies recognize CTD autoantigens. Line plots display MFI levels of antibodies targeting traditional autoantigens that are inducible (SRP54 in individual AA19; TPO in individual AA23), fluctuate (TPO in individual AA13), or do not change significantly over time (most individuals with TPO autoantibodies, and 2 individuals with anti–PDC-E2).

The Athens influenza cohort included longitudinal serum samples, enabling assessment of ACA and CTD-AAbs trajectory over the course of illness, with up to 1 month of follow-up for a subset of patients. Of the 31 patients with influenza with at least 2 samples available, 13 (41.9%) had ACA and 8 had CTD-AAbs in baseline samples (Figure 3, A and B, and Supplemental Figure 1). For available samples after 1 week, autoantibody-positive MFI levels remained elevated (within 50% of baseline or increased) in 10 of 12 (83%) patients for ACA and 6 of 7 (86%) patients for CTD-AAbs. At 4 weeks, autoantibody-positive MFI remained elevated in 6 of 8 (75%) patients for ACA and 5 of 7 (71%) patients for CTD-AAbs.

We observed emergence of CTD-AAb in 2 patients (Figure 3B). Autoantibodies recognizing SRP54, a myositis-associated autoantigen, increased by over 4-fold between the first and second time points in individual AA19 who also carried anti–PDC-E2 and anti–TPO CTD-AAbs at baseline. Anti-TPO developed at the 1-month time point in individual AA23. These data reveal that a subset of patients acutely infected with influenza develop autoantibodies that were undetectable at baseline.

### A subset of ACA has functional receptor blocking activity.

**Figure 4:**
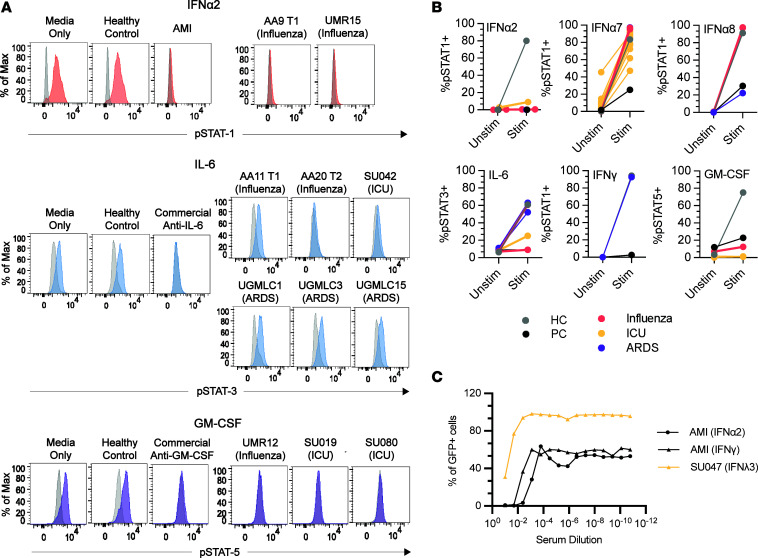
Cell-based cytokine receptor-blocking assays. (**A**) FACS plots of IFN-α2, IL-6, and GM-CSF signaling assays. Cells were treated with media only; commercial blocking antibody or 10% positive control serum from a patient with atypical mycobacterial infection (AMI); 10% healthy control serum; or 10% test serum. Cells were treated with patient serum or a control in the unstimulated condition and with both cytokine and patient serum or a control in the stimulated condition. (**B**) Blocking activity of patient serum on cells in cytokine signaling assays, reported as percentage of pSTAT^+^ cells in the unstimulated and stimulated condition. Patient sera were from patients with influenza (*n*_Marburg_ = 4, *n*_Athens_ = 5), Stanford ICU (*n*_infected_ = 19, *n*_noninfected_ = 2), or ARDS (*n* = 8) . For IFN-α2 and IFN-α8, results shown represent 2 independent experiments (Supplemental Figure 7). HC and positive controls (PC; commercially available antibody or prototype patient serum with known blocking activity) are also included. (**C**) Neutralization activity to IFN-α2, IFN-γ, and IFN-λ3 in the serum samples of 2 patients. IFN-α2, IFN-γ, and IFN-λ3 were incubated with heat-inactivated serum from donor AMI (PC) and donor SU047 (infected Stanford ICU cohort) and added to HAP1 reporter cells. The serum samples were prepared and tested with a 5-fold serial dilution on HAP1 reporter cells. Final concentrations of IFN-α2, IFN-γ, and IFN-λ3 in the culture were 40 U/mL, 8 U/mL, and 1 ng/mL, respectively (Supplemental Figure 8). The percentages of GFP^+^ HAP1 reporter cells were evaluated 22–24 hours after the incubation with flow cytometry.

We used cell-based cytokine blocking assays to assess whether patient sera with high MFI values for ACA inhibit receptor signaling in vitro (Figure 4). For each cytokine blocking assay, patient sera were selected based on an MFI value > 5 SD above the HC average and > 3,000 MFI. A total of 39 samples met these criteria and were available for assessment of blocking activity (Stanford ICU, *n*_infected_ = 20, *n*_noninfected_ = 2; ARDS, *n* = 8; Giessen/Marburg influenza, *n* = 4; and Athens influenza, *n* = 5). From pSTAT induction blocking assays, blocking activity for at least 1 cytokine was observed in 11 of 39 ACA^+^ samples analyzed across the 5 cohorts (28%, see Table 2 for summary and clinical characteristics). Complete or partial blocking of STAT1 phosphorylation was observed in all 3 anti–IFN-α2^+^ samples (Figure 4A), 1 of 22 anti–IFN-α7^+^ samples, and 1 of 3 anti–IFN-α8^+^ samples (Figure 4B). Complete or partial blocking was also observed in 2 of 6 anti–IL-6^+^ samples and in all 3 anti–GM-CSF^+^ samples, 2 of which were Stanford ICU samples and 1 of which was an influenza sample (Figure 4, A and B). Neither patient was taking anti–IL-6 therapies. We also detected blocking activity from an anti–IFN-λ3^+^ serum sample using a GFP reporter cell line (Figure 4C).

Blocking activity was detected in at least 1 sample from all cohorts. Five of the 6 Stanford ICU blocking samples were derived from patients without clinical evidence of viral infection (Table 2). Samples with blocking activity did not always correspond with the highest MFI values for the relevant ACA, consistent with observations in COVID-19 (10). As reported in severely ill patients with COVID-19, none of the anti–IFN-γ^+^ samples had blocking activity (Figure 4B, bottom left panel). We conclude that a subset of ACA block binding to their cognate receptors, even in patients infected with bacterial pathogens not previously linked to defects in these cytokine signaling pathways.

## Discussion

We previously demonstrated that ~25% of hospitalized patients infected with SARS-CoV-2 develop newly detectable IgG autoantibodies that recognize cytokines and autoantigens typically associated with CTDs, such as myositis, systemic lupus erythematosus (SLE), and SSc (5). A critical unanswered question in these studies is whether autoantibodies are triggered in other acute infections and, if so, whether the target antigens differ from those identified in COVID-19. Here, we report that ACA and CTD-AAbs are indeed found across a spectrum of patients with non–SARS-CoV-2 infections, including infections caused by viral pathogens and known or suspected bacterial pathogens. Pulmonary and nonpulmonary infections were associated with ACA, suggesting that secreted proteins are targeted across a spectrum of organ systems. Although the differences were not statistically different, autoantibodies specific for CTD antigens were more prominent in patients with infections than in those thought to be uninfected, and they were markedly higher than levels seen in HC. Longitudinal data suggest that, while most of these autoantibodies are present at the time of presentation, some can emerge over time and can persist for at least 28 days after infection.

A key advance presented here is the widespread nature of autoantibodies that are seen across not only multiple respiratory viral infections, but also nonrespiratory bacterial infections observed in patients admitted to the ICU. A strength of our study is the availability of rich clinical data on all cohorts, enabling correlations between clinical data and autoantibody profiles. In the Stanford ICU cohort, ~25% of the population was clinically phenotyped as uninfected by 3-physician review yet still had substantial rates of autoantibody elevation in comparison with HC. It is important to note that clinical phenotyping for infection is known to be imprecise, with a cultured organism present only ~40%–60% of the time (11–13). Some of the Stanford ICU patients were admitted with exacerbations of chronic obstructive pulmonary disease or asthma, and it is possible that viral infections or bacterial superinfections preceding admission were not identified. Cohorts from Marburg, Giessen, and Athens were also deeply phenotyped, including clinical and laboratory testing, the identification of specific pathogens, clinical outcomes, and vaccination status.

Anti–Type I IFN antibodies have been a major focus of autoantibody studies, particularly those on COVID-19 (6, 10, 14, 15). Anti–Type I IFN are prevalent in severe COVID-19 but not in asymptomatic or mildly ill patients with SARS-CoV-2 infections. Multiple publications have demonstrated that a subset of anti–Type I IFN IgG antibodies block binding to the IFN-α/β receptor (IFN-AR), prevent activation of JAK/STAT signaling pathways, and facilitate viral replication in in vitro, cell-based models (3, 14, 16, 17). A causal link between preexisting anti–Type I IFN and pathogenesis is suggested by the increased mortality observed in SARS-CoV-2–infected patients with autoimmune polyglandular syndrome type 1 (APS-1) (10) and poorly controlled infection in patients exposed to the live, attenuated yellow fever virus vaccine (15). Our study identified 1 individual with blocking anti–IFN-α2 in 2018 who presented 2 years later with multiple severe viral infections including SARS-CoV-2, suggesting lasting susceptibility to viral infection (Table 2). It remains unclear whether anti–Type I IFN remain as fixed components of the autoantibody repertoire of severely ill patients with COVID-19 and whether they might predispose such patients to superinfection or subsequent severe infection with other pathogens (18).

Here, we show that anti–Type I IFN are also frequently found in ICU patients (Supplemental Figure 2), particularly in patients with infections compared with those who appear to be uninfected (Figure 1B). We identified 5 patients with anti–Type I IFN blocking activity (Table 2 and Figure 4). Consistent with previous reports, all were over the age of 60, and all were male. A female patient (individual SU008) had IFN-α7–neutralizing antibodies and developed ARDS following infection with respiratory syncytial virus (RSV). Individual UMR15 had IFN-α2–neutralizing antibodies and developed severe influenza at age 66. Two years later, the same individual developed severe COVID-19 and ARDS, requiring mechanical ventilation 2 weeks after the first COVID-19 mRNA vaccination and complicated by the reactivation of cytomegalovirus (CMV) and herpes simplex virus (HSV) pneumonitis. Although preinfection samples are unavailable for other patients analyzed in this report, a subset of patients who develop symptomatic influenza or other respiratory infections most likely harbor preexisting ACA that predispose them to develop ARDS when infected with more virulent pathogens, such as SARS-CoV-2. Because blocking ACA can be found even in patients with undetectable IgG autoantibodies by ELISA or bead-based assays used here, our results likely underestimate the true prevalence of functional ACA in patients with non–SARS-CoV-2 infections (14, 19). Future studies will be needed to understand the risk for superinfection as well as the efficacy of vaccines in patients with blocking ACA, including determining whether ACA such as anti–Type I IFN are enriched in patients with breakthrough influenza or SARS-CoV-2 infections.

Many non–IFN-α ACA were identified in these cohorts, including antibodies specific for ILs (IL-2, IL-17A, and IL-22) and TNF-α (Figure 1B), and less frequent targets such as IFN-γ, IL-6, IL-12p70, GM-CSF, and sRANK ligand (Figure 2). We identified IgG blocking activity for at least 1 sample for 3 different Type I IFNs, IL-6, GM-CSF, and IFN-λ (Figure 4 and Table 2). Blocking ACA have been identified in multiple immunodeficiency disorders (17, 20), SLE (21, 22), COVID-19 (3, 6), atypical infections (7), and a variety of other diseases (23).

Even if only a minority of ACA are found to have in vitro blocking activity, their high prevalence in patients with COVID-19 and these cohorts suggests that nonblocking ACA may still play a role in disease pathogenesis (Figure 1B). One potential mechanism for this could be an increase in nonblocking ACA levels via activation of pre-existing autoreactive B cells, driven by local production of the targeted cytokine that serves as an autoantigen. The resulting immune complex could indirectly inhibit local cytokine binding to its receptor, reducing downstream signaling and enhancing pathogen replication and/or inflammation. If correct, this model would have important therapeutic implications. For example, patients with anti–IFN-α antibodies could benefit from treatment with exogenous IFN-β, which also binds and activates IFN-AR and has not been identified as a prominent autoantigen in this study or in COVID-19 (5).

One of our most striking findings is that some patients infected with influenza develop newly detectable autoantibodies, an observation we recently described in hospitalized patients with COVID-19 (5). Autoantibodies including anti-SRP54 and anti-TPO developed in 2 patients with influenza and remained elevated approximately 1 month after their first hospital visit, suggesting that viral infection triggered autoantibody development. Anti-SRP54 is associated with immune-mediated necrotizing myopathy (IMNM) and is a recognized biomarker for myositis. Multiple reports have linked prior infection to the onset of anti-SRP myositis (24, 25). The role of anti-SRP54 in myositis and how anti-SRP54 may be induced during infection, however, remains unknown.

Many other infectious agents have been linked epidemiologically and molecularly to the subsequent development of autoimmunity, including pandemic influenza (26), Epstein Barr Virus (SLE and multiple sclerosis; refs. 27–30), and dengue virus (antiplatelet antibodies and thrombocytopenia; ref. 31). When considering these well-described examples with published studies on COVID-19 and our current report, it appears that the potential infectious agents have for triggering specific autoantibodies may be much higher than previously recognized.

The mechanisms by which tolerance to self-antigens is broken, even if transiently, in COVID-19, influenza, and other infections is largely unknown. Molecular mimicry has been widely proposed in COVID-19 studies, with over 100 PubMed citations to date citing this mechanism; however, no convincing studies have yet to demonstrate this experimentally. Many ACA are detectable at the time of infection, and their levels appear to remain mostly constant (e.g., anti–IFN-γ and anti–IFN-α2) or increase modestly (e.g., anti–IFN-α7; Figure 3A) over time. Thus, molecular mimicry is unlikely to explain the large increases in levels of newly detected ACA previously described in severely ill patients with COVID-19, such as inducible ACA recognizing IL-22, IL-17, and IFN-ε (5). Molecular mimicry is also unlikely to explain the development of anti-SRP54 and anti-TPO in influenza and SARS-CoV-2 infection (Figure 3B), as this mechanism would imply that self-proteins cross-react with proteins from 2 unrelated respiratory viruses. Finally, autoimmune thyroiditis and anti-TPO are commonly observed following transplantation, cancer treatment with checkpoint inhibitors, and in many autoimmune diseases; this information can be used to argue against an infection-specific mechanism.

A more likely explanation is that cytokines and IFNs secreted in response to viral infection drive ACA production by preexisting autoreactive B cells. We previously reported that patients with APS-1 with serum anti–Type I IFN and IL-17A display in their blood an accumulation of autoreactive mature naive B cells, some with measurable reactivity to Type I IFN and IL-17A (20). Hence, early impairments of naive B cell selection, associated with many autoimmune patients, may contribute to the production of ACA-expressing B cells and secretion of ACA, a response that may be enhanced during infection (32). It remains to be determined whether potential defects in early B cell tolerance checkpoints in ICU patients with ACA result from genetic alterations, such as autoimmune regulator (AIRE) deficiency in patients with APS-1, promoting sustained serum ACA over time, or if these B cell tolerance defects are only transiently induced during infection.

Finally, autoantibodies are postulated to play a role in a subset of COVID-19 survivors with “long-haul” symptoms (termed postacute sequela of COVID-19 [PASC]) that have been well described and are under active study (2, 33). Many PASC characteristics mirror the known long-term effects of sepsis and critical illness (often termed post-ICU syndrome [PICS]), with a substantial proportion of patients still reporting diminished quality of life or new-onset neurologic and psychiatric deficits 6 and 12 months after acute illness (34). Survivors of ARDS similarly report diminished functional status even 1 and 5 years after discharge, despite lung function returning to near-normal levels (35). Data on the duration and clinical implications of autoantibodies in longer-term recovery of patients with PASC, PICS, and critical illness are lacking in the cohorts of this report. Whether these lingering symptoms reflect persistent autoimmunity or inflammation requires further study.

Our study has several limitations that require future work. The number of individuals characterized is relatively small, and some patients who were described as “noninfected” may have been infected and asymptomatic. Moreover, only 1 patient cohort was available for longitudinal analysis to assess durability of autoantibody elevation. It is unknown whether a subset of patients with autoantibodies present early in the course of disease will go on to develop CTD autoantibody–associated clinical manifestations such as myositis (e.g., anti-SRP54) or thyroiditis (anti-TPO). Additionally, the impact of the presence of autoantibodies on outcomes is not clear in these cohorts. Because the vast majority of patients in these cohorts had severe disease, an association with severity of illness and, thus, outcomes would be missed. Except for 1 influenza-infected patient who later developed severe COVID-19, preinfection samples were unavailable to definitively determine whether autoantibodies predated infection. Although the bead-based array platform used in our studies is approved for use at some academic centers for Clinical Laboratory Improvement Amendments–level (CLIA-level) testing, our arrays have been developed and widely used only for research purposes and have not been directly compared with clinical-grade assays. Finally, we do not have paired PBMCs to correlate our autoantibody findings with analyses of immune cell populations, particularly to explore defects in autoreactive B cell subsets.

In summary, the scale of the COVID-19 pandemic and the availability of well-annotated, longitudinally collected biospecimens has advanced our understanding of how the balance between direct viral injury and triggered inflammatory response contributes to the wide spectrum of disease severity. The studies described in this report significantly extend discoveries in COVID-19, revealing a high prevalence of autoantibodies in serum of patients with non–SARS-CoV-2 viral, bacterial, and fungal infections. Future experiments on larger cohorts of outpatients are needed to determine whether our results extend into an ambulatory setting and the longer-term duration and clinical implications of these autoantibodies. Understanding the underlying immunologic mechanisms of autoantibody formation could lead to a transformation in approach to acute infection, with a focus not only on the pathogen, but also on the triggering of an autoimmune disorder in a subset of patients.

## Methods

### Serum and plasma samples.

Serum or plasma samples were obtained following informed consent and cryopreserved at –80°C until antibody profiling was performed.

### Stanford ICU patient samples.

The Stanford ICU biobank is a collection of whole blood samples prospectively obtained from individuals admitted to Stanford Hospital ICU with at least 1 risk factor for ARDS (e.g., sepsis, aspiration, and/or trauma). Exclusion criteria included routine postoperative patients and severe anemia. Clinical data were abstracted from the medical record by study staff blinded to autoantibody levels. Infection status was determined through retrospective chart review by 3 physicians blinded to autoantibody levels. Positive or negative infection status was defined by a consensus of at least 2 of 3 physicians as previously described (13). Samples were collected between February 2015 and November 2018, and all patient samples and data collected were compliant with the Stanford IRB (*n* = 167, Stanford IRB no. 28205; Supplemental Table 1).

### Giessen and Marburg acute respiratory illness patient samples.

Serum samples were obtained from hospitalized individuals at 2 academic centers in Germany (Giessen and Marburg). Serum samples from patients admitted to the ICU between April and May 2020 for COVID-19 symptoms but who tested negative by PCR (*n* = 19) and serum samples from patients hospitalized with Influenza A and pneumonia between January 2018 and March 2020 (*n* = 25) were obtained from the Philipps University Marburg (IRB no. 57/20). Serum samples from patients with ARDS (*n* = 17) were obtained from the University of Giessen and were collected between July 2016 and January 2020 (IRB no. 58/15) See Supplemental Tables 2–4 for details. Using a viral array recently described by our group (5), we identified 1 patient who tested negative for SARS-CoV-2 based on PCR but was positive for antibodies against SARS-CoV-2 spike protein (Supplemental Figure 6). This patient was excluded from subsequent analyses.

### Acute influenza infection patient samples.

Patients were admitted to the Sotiria Thoracic Diseases Hospital of Athens (approval no. 16707/10-7-18) and the Attikon University Hospital of the University of Athens Medical School (approval no. 1821A/22-9-16) in Greece between December 2018 and April 2019 and were diagnosed with acute influenza by the BioFire FilmArray Respiratory Panel (RP) test (bioMerieux, RFIT-ASY-0124) (*n* = 40). Serum samples were collected from each patient at 1–3 time points. Samples from the first time point (T1) were collected on the day that the patient visited the hospital and diagnosed with influenza. Samples from T2 were collected approximately a week later. Samples from T3 were collected approximately a month after the initial visit. Serum was collected at T1 for all 40 patients, while serum was collected at T2 and T3 for 29 and 20 patients, respectively (Supplemental Table 5).

### COVID-19 patient samples.

Serum samples that had been characterized previously using protein arrays were obtained from hospitalized patients with COVID-19 from Philipps University Marburg between April and June 2020 (*n* = 18, IRB no. 57/20) (5). Two samples with high levels of ACA were selected to develop blocking assays.

### HC samples.

Serum and plasma samples from anonymous HC (*n* = 33) were obtained prior to the COVID-19 pandemic from Stanford Blood Bank and Stanford Hospital and Clinics. Normal human sera (ImmunoVision, HNP-0300, certified to be nonreactive to Hep-2 cell lysates at a titer of 1:100) was used for validation and as negative controls in array experiments.

### Positive control individuals with known autoimmune disease and known blocking autoantibodies.

Prototype human plasma samples derived from participants with autoimmune diseases with known reactivity patterns (e.g., topoisomerase 1 [Scl-70], centromere, Sjögren’s Syndrome type A [SSA], SSB, whole histones, and ribonucleoprotein [RNP]) were purchased from ImmunoVision or were obtained from Stanford Autoimmune Diseases Biobank and Oklahoma Medical Research Foundation (Oklahoma City, Oklahoma, USA; a gift of Judith James, Oklahoma Medical Research Foundation). Serum from patients with APS-1, IPEX, PAP, and AMI (17) were provided by David Lewis (Stanford) and used for array experiments and for blocking studies.

### Bead-based antigen arrays.

Two different custom, research-grade, bead-based antigen arrays were created, as previously described (20, 21, 36–40). A complete list of all antigens, vendors, and catalog numbers can be found in Supplemental Tables 6 and 8. Arrays were constructed as previously described. Briefly, antigens were coupled to carboxylated magnetic beads (MagPlex-C, Luminex Corp.), each with unique barcodes (36, 37). Immobilization of some antigens and control antibodies on the correct bead IDs was confirmed using commercially available mouse monoclonal antibodies or antibodies specific for engineered epitope tags. Prototype human plasma samples were used for validation of bead arrays.

### Array probing.

Serum or plasma samples were tested at 1:100 dilution in 0.05% PBS-Tween supplemented with 1% (w/v) BSA and transferred into 96-well plates in a randomized layout. The bead array was distributed into a 384-well plate (Greiner BioOne) by transfer of 5 μL bead array per well. A total of 45 μL of the 1:100 diluted sera was transferred into the 384-well plate containing the bead array. Samples were incubated for 60 minutes on a shaker at room temperature. Beads were washed with 3 × 60 μL PBS-Tween on a plate washer (EL406, Biotek), and 50 μL of 1:1,000 diluted R-phycoerythrin–conjugated (R-PE–conjugated) Fc-γ–specific goat anti–human IgG F(ab’)2 fragment (Jackson ImmunoResearch, 106-116-098) was added to the 384-well plate for detection of bound human IgG. After incubation with the secondary antibody for 30 minutes, the plate was washed with 3 × 60 μL PBS-Tween and resuspended in 50 μL PBS-Tween prior to analysis using a FlexMap3D instrument (Luminex Corp.). Binding events were displayed as MFI. All samples were run in duplicate in each experiment. Longitudinal samples that showed new-onset autoantibodies were reanalyzed in duplicate on new bead arrays to confirm results. Samples from patients with COVID-19 were heat inactivated prior to analysis, as previously described (41).

### pSTAT induction in cell-based assay.

The blocking activity of patient sera with specific ACA was assessed as previously described (3). Cells (400,000 cells/condition) were incubated with 10% HC serum, patient serum or plasma, commercial blocking antibody, positive control blocking serum, or media only for 15 minutes and stimulated with the appropriate cytokine. The percentage of pSTAT^+^ cells was compared between the stimulated and unstimulated condition. To develop each assay, cells were stimulated at different cytokine concentrations to determine the final working concentration. The lowest concentration at which maximal stimulation was observed was selected for the final blocking assays (Supplemental Figure 7). Patient sera positive by array for anti–IFN-α2, –IFN-α7, –IFN-α8, –IFN-γ, –IL-6, and –GM-CSF antibodies were assessed using blocking assay conditions and U937 (ATCC CRL1593) and THP-1 (ATCC TIB-202) cell lines, as summarized in Supplemental Table 9. Cells were assessed on a BD LSR II analyzer and analyzed using FlowJo software version 10.8. A complete list of cytokines, blocking antibodies, staining antibodies, vendors, and catalog numbers can be found in Supplemental Table 10.

### GFP reporter assays.

The activities of IFN-α2, IFN-γ, and IFN-λ3 were detected by a HAP1 reporter cell line received from Jan Carette at Stanford (42). This cell line expresses GFP under the control of the IFN-stimulated response element (ISRE) of IFN-induced protein with tetratricopeptide repeats 2 (*IFIT2*) and is sensitized for IFN-λ detection by stable overexpression of *IFN-LR1* (42). HAP1 reporter cells were cultured with complete IMDM containing 10% FBS and 1% penicillin/streptomycin/amphotericin. HAP1 reporter cells were seeded into 48-well plates with 3 × 10^4^ cells per well and incubated overnight. To evaluate the function of HAP1 reporter cells of indicating the activities of IFNs, IFNs were prepared into a 5-fold serial dilution in serum-free IMDM and added to the HAP1 reporter cells (Supplemental Figure 8A). The cells were incubated 22–24 hours at 37°C with 5% CO_2_, treated with 0.25% trypsin, suspended into single cells, and analyzed by flow cytometry (Cytek Aurora) for the expression of GFP. To evaluate the neutralization activity of monoclonal antibodies to each IFN-, the antibodies were prepared by a 2-fold serial dilution in serum-free IMDM (Supplemental Figure 8, B and C). To evaluate the neutralizing activity in the serum samples, serum was heat-inactivated at 56°C for 30 minutes and prepared into a 5-fold serial dilution. The serial-diluted monoclonal antibodies and serum were cultured with their cognate IFNs for 1 hour at room temperature. The concentrations of IFN-α2, IFN-γ, and IFN-λ3 during the incubation were 80 U/mL, 16 U/mL, and 2 ng/mL, respectively. The IFN-antibody and IFN-serum mixtures were then added into HAP1 reporter cells, and the final concentrations of IFN-α2, IFN-γ, and IFN-λ3 in the cell culture were 40 U/mL, 8 U/mL, and 1 ng/mL, respectively. GFP expression in the cells was evaluated 22–24 hours after incubation as described above. A complete list of the cytokines and monoclonal antibodies used, and their vendors and catalogs, can be found in Supplemental Table 11. The activities of IFN-α2, IFN-γ, and IFN-λ3 were evaluated by measuring the percentages of GFP^+^ HAP1 reporter cells. The reduction of GFP signal due to the blocking activity of monoclonal antibodies or patient serum was calculated by subtracting the background signal (the percentage of GFP^+^ cells in the cell culture without any treatment) and then dividing by the maximal cytokine-induced signal (the percentage of GFP^+^ cells when cells were cultured with IFN alone). The reduction of GFP signal for each condition was plotted, and 4-parameter inhibitory dose-response curves were fitted to the data using GraphPad Prism v.9.3.0. The half-maximal inhibitory concentration (IC_50_) was calculated using the equation,


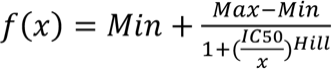
, (1)

where f(x) is the reduction of GFP signal, x is the concentration of the antibody in pg/mL, “Min” and “Max” are the plateau values of the Y axis, “Hill” is the Hill coefficient, and IC_50_ is the concentration of antibody where the reduction of GFP signal is halfway between the “Min” and “Max” values.

### Data and code availability.

All raw and normalized array data are publicly available on the Gene Expression Omnibus (GEO) database with the accession no. GSE222765. Code used for data analysis and figure generation are available from the corresponding authors upon request.

### Statistics.

R, RStudio, and various R packages were used to perform analyses (43, 44). For normalization, MFI values for “bare bead” IDs were subtracted from MFI values for antigen-conjugated bead IDs, and replicate MFI values were averaged. The average MFI for each antigen was calculated using samples from healthy individuals (all obtained before December 2019). Serum samples were considered “positive” for antibodies recognizing a specific antigen if the normalized MFI was > 5 SD above the average MFI for HC for that antigen and if the normalized MFI was > 3,000 units, a more stringent threshold than those commonly published in related literature (3). Based on the SAM algorithm (45), statistically significant antigens were identified using FDR-adjusted *P* values (*q* < 0.001), 2-fold change cutoffs, and 10,000 permutations. Statistical differences in MFI between groups for each antigen were determined using 2-sided Wilcoxon rank-sum tests with Bonferroni correction. *P* values less than 0.05 were considered significant. Data were visualized in GraphPad Prism v.9.3.0. Complexheatmap v.2.8.0 was used for all heatmaps (46). Upon publication of this study in a peer-reviewed journal, deidentified array data will be uploaded to the Gene Expression Omnibus (GEO) database (https://www.ncbi.nlm.nih.gov/geo/query/acc.cgi?acc=GSE222765).

Within the Stanford ICU cohort, analyses evaluated predictors for the development of autoantibodies as well as pertinent ICU outcomes related to the presence of autoantibodies in patient serum. Fisher’s exact test was used to evaluate the association between age (< 60 versus ≥ 60), sex, immunocompromised status, WBC count (< 12,000 versus ≥ 12,000/μL), and infection status with the development of ACA and CTD-AAb. To control for interactions between variables, 2 adjusted models were performed using logistic regression analysis. We adjusted for baseline characteristics (sex, age, and race) and performed a fully adjusted model using baseline characteristics in addition to presence of shock (pressors at day 0) and APACHE II score as predictors for the presence or absence of ACA and CTD-AAb.

### Study approval.

Serum samples from all 4 centers were obtained following informed consent and with each institution’s IRB approval.

## Author contributions

AF, EYY, SEC, SD, PJU, and CS designed experiments and array panels. AF, EYY, SEC, and SD performed antigen array and ACA array production, quality control, sample runs, and data acquisition. GS assisted PJU with supervision of AF, EYY, XY, and SD and with data analysis and interpretation. RP, ML, MS, KP, JHL, CMR, JEC, and CAB developed the IFN-λ neutralizing antibody screening assay using HAP1 reporter cell lines. MG, EKMM, CS, PCF, SV, SH, VT, and MLS collected and processed patient blood samples. MG, CS, EKMM, SH, PCF, ST, VT, and EA collected, extracted, and analyzed clinical data. EKMM, AN, HR, RG, SH, BS, CS, PCF, ST, and EA assisted in designing, recruiting, and/or following inpatient and HC cohorts. ARM, BS, SH, VT, MLS, EA, JGW, JEL, AAA, WB, US, and KCN supervised clinical data management and biobank recruitment and/or performed chart reviews that enabled correlation of array results with clinical parameters. BS, WB, and JR contributed to collection and storage of patient samples, collection of patient laboratory and clinical data, and distribution of blood samples. AF, BS, WB, EYY, RP, CAB, SD, SH, KCN, ARM, AJR, XY, JF, CS, ETLP, and PJU analyzed data, performed statistical analyses, and/or interpreted data across experiments and cohorts. AF, EYY, SD, XY, ARM, RP, AJR, CS, and PJU created figures and tables. AF, EYY, ARM, SD, CS, AJR, and PJU were primary authors of the manuscript. AF, EYY, ARM, SD, RM, EM, TTW, ETLP, CS, AJR, and PJU contributed significantly to editing the manuscript, interpreting results, and drafting relevant sections in the Discussion. PJU, CS, CAB, EA, and AJR supervised the research. Most of the design, data generation, data analysis, interpretation, and drafting of the manuscript was a deep, collaborative effort between the 4 first authors. We have read the recent *JCI* editorial by Casadevall (129:2167, 2019) and have carefully considered the order of authorship, which was largely agreed upon early in the process of experimental design and modified as the project proceeded. AF was co–first author (second-named author) of the original *Nature Communications* study on new-onset autoantibodies in COVID-19. He performed most of the data analysis for that study, while the named first author (SC) — who contributed to analysis and performed almost the same array experiments as AF (and all other Stanford undergraduates) — was not allowed to work on the Stanford campus for the better part of the year. The other 2 named first authors were from the University of Pennsylvania. Overall, the first named author is a woman, and 2 of 4 first authors are women. Two of the 3 senior and corresponding authors were women. In this current *JCI Insight* submission, AF is the named co–first author. He worked on the project from the beginning and, at the time, was the only scientist in the lab (SEC had moved to Genentech, SD to medical school). EYY is the second-named co–first author. She joined the lab in summer 2021 and participated in running anti-cytokine cell-based assays, interpreting the data, generating figures, and writing a significant portion of the manuscript, including the Discussion. ARM is a pulmonology fellow who performed analysis of the associated clinical data for the Stanford cohort, under the direction of AJR. He wrote on these findings as well as significant portions of the Discussion. Finally, SD performed array experiments and designed, developed, and ran many of the cell-based cytokine blocking experiments. He drafted those figures, assisted with writing, and was actively involved in editing prior to leaving for medical school. Two of the 3 senior and corresponding authors are women.

## Supplementary Material

Supplemental data

## Figures and Tables

**Table 1:**
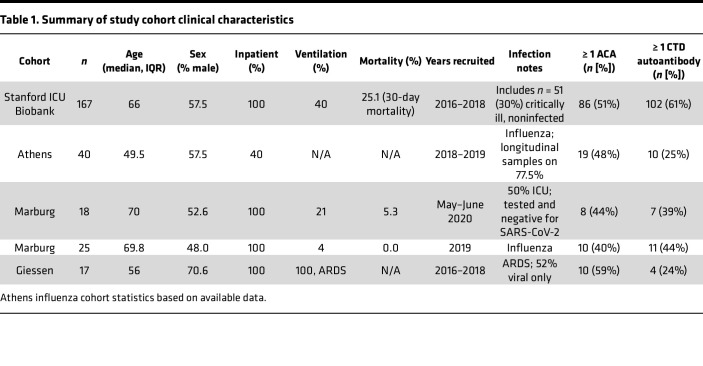
Summary of study cohort clinical characteristics

**Table 2:**
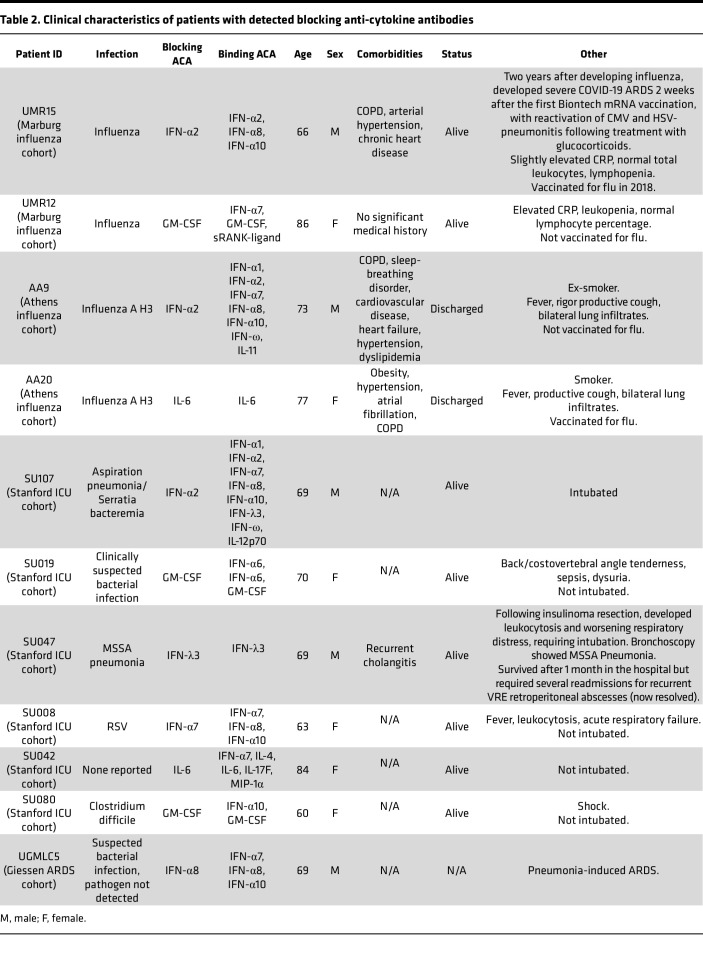
Clinical characteristics of patients with detected blocking anti-cytokine antibodies

## Version 1 02/08/2023

Electronic publication
