# Supplementary material for: Autoantibodies are highly prevalent in non–SARS-CoV-2 respiratory infections and critical illness
Source: JCI Insight. 2023 Feb 8;8(3):e163150. doi: 10.1172/jci.insight.163150 (PMC9977421; doi:10.1172/jci.insight.163150)
Supplement: Supplemental data [file jciinsight-8-163150-s125.pdf]

SUPPLEMENTAL FIGURES

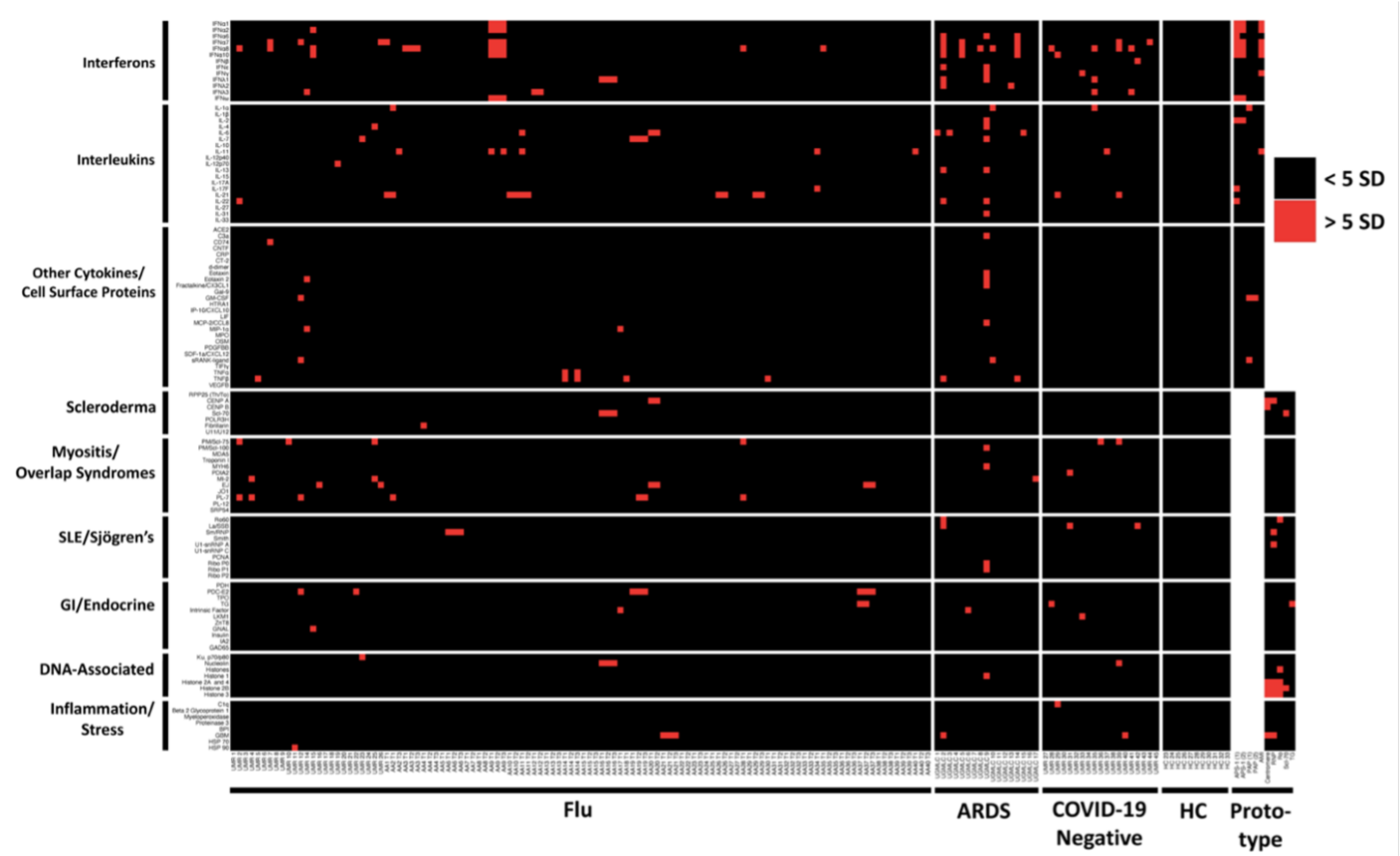

Supplemental Figure 1: Autoantibodies in ICU patients and patients with influenza. Heatmap representing serum IgG antibodies measured in influenza patients (n = 65); ARDS patients (n = 17), both collected prior to the COVID-19 pandemic; ARDS patients who tested negative for COVID-19 (n = 19); and HC (n = 11) using a 58-plex cytokine array (upper panels) and 55-plex traditional CTD autoantigen array (lower panels). Prototype serum samples (n = 5 on both arrays) were also tested. Cytokines are grouped on the y-axis by category (interferons, interleukins, and other cytokines/growth factors/receptors). Connective tissue disease antigens are grouped on the y-axis by disease category (scleroderma, myositis and overlap syndromes such as mixed connective tissue disease (MCTD), SLE and Sjögren's, gastrointestinal and endocrine disorders); association with chromatin; and association with tissue inflammation or stress responses. Colors indicate autoantibodies whose MFI measurements are >5 SD (red) or <5 SD (black) above the average MFI for HC. MFIs <3,000 were excluded.

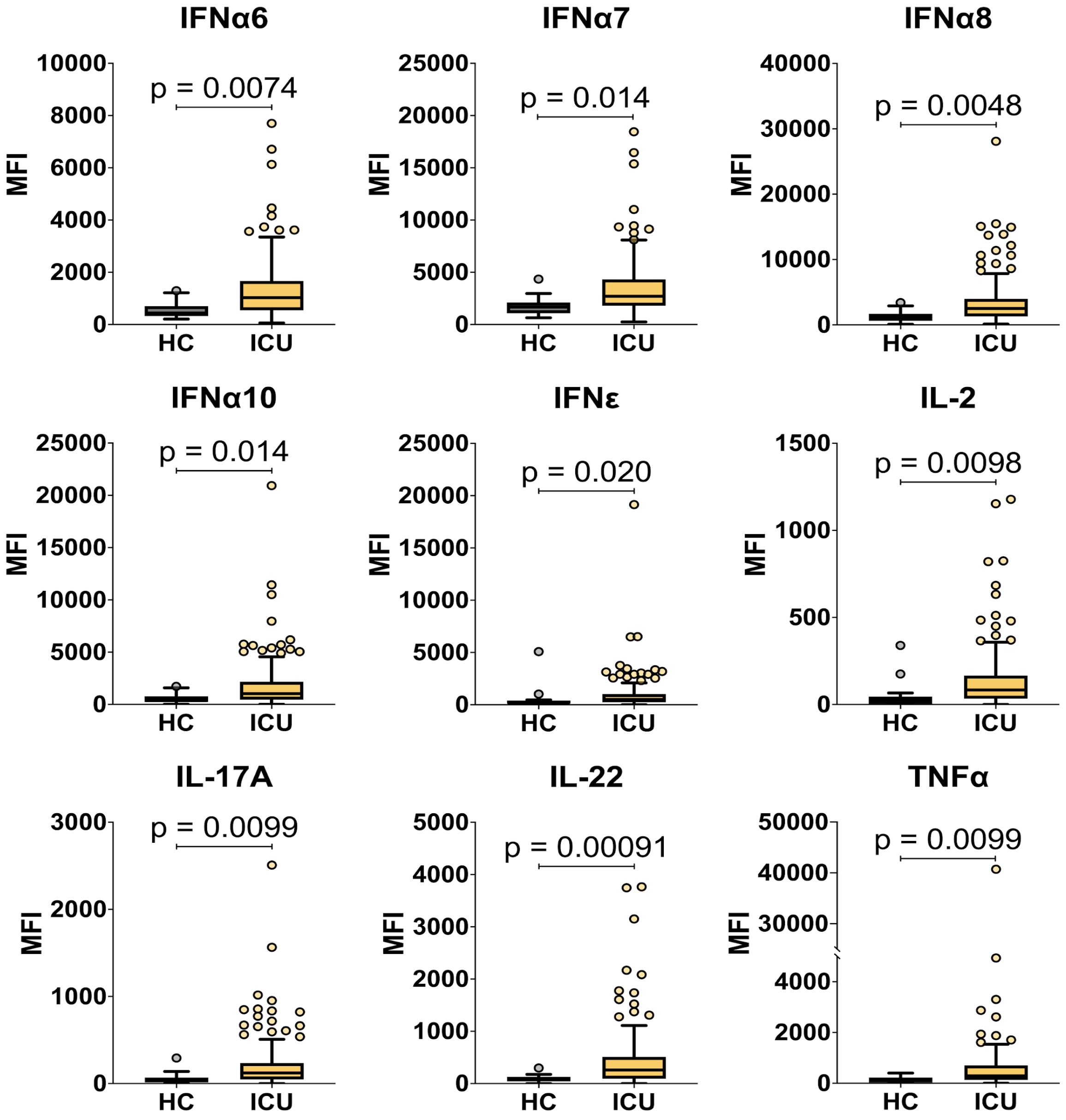

Supplemental Figure 2: High prevalence of ACA in hospitalized ICU patients compared to HC regardless of infection status. Tukey box plots comparing MFI data from Stanford ICU patients (n = 167) and HC (n = 22) for nine antigens for which statistically significant differences were determined between ICU patients (regardless of presence or absence of infection) and HC using two-tailed Wilcoxon rank-sum tests with Bonferroni correction. The middle line represents the median, while the lower and upper hinges correspond to the first and third quartiles. The upper whisker extends from the hinge to 1.5 times the interquartile range (IQR) above the 75th percentile MFI value, and the lower whisker extends from the hinge to 1.5 times the IQR below the 25th percentile MFI value.

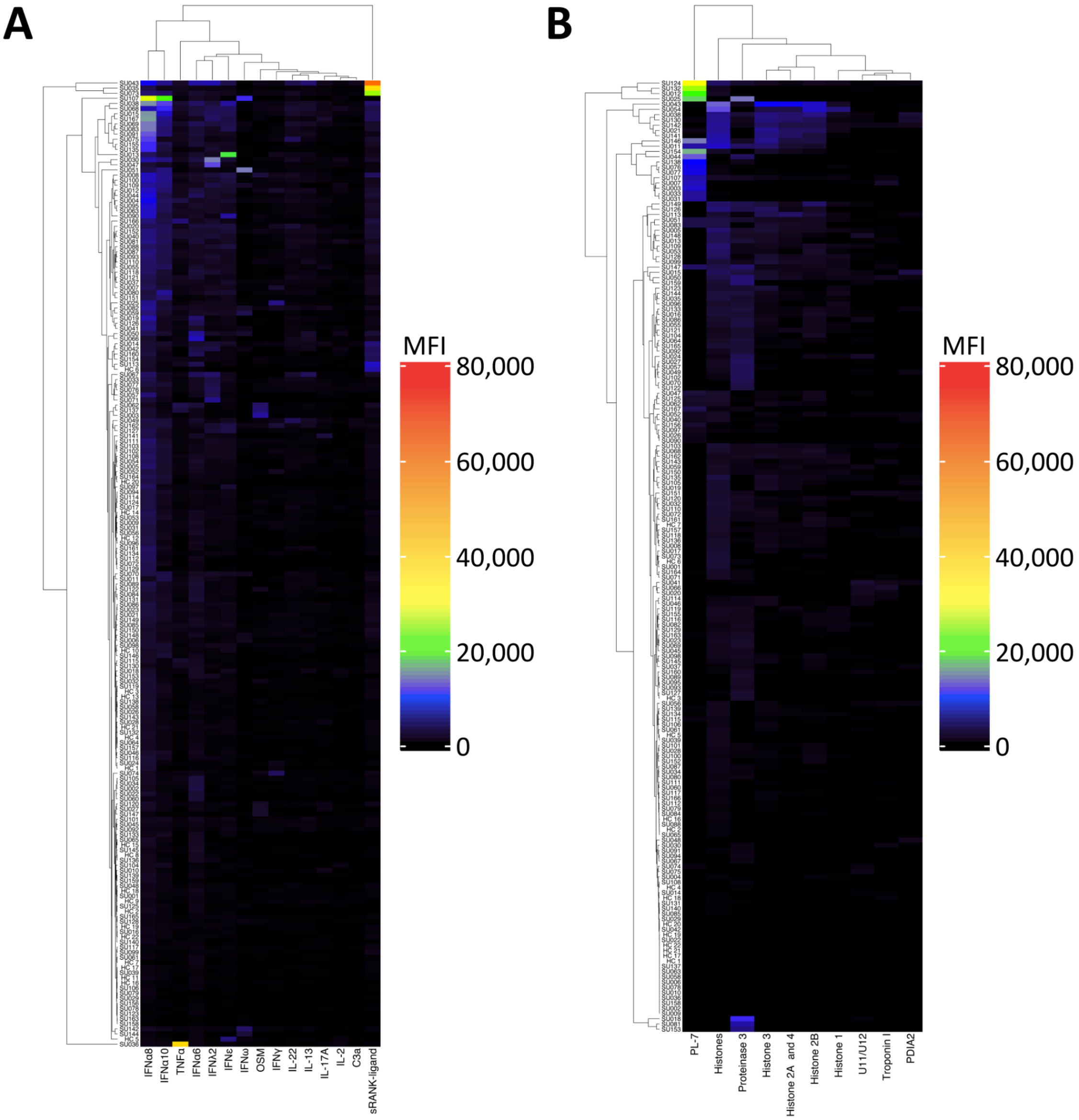

Supplemental Figure 3: Array analysis of Stanford ICU patients using the SAM algorithm. Clustered heatmap representing antigens with statistically significantly higher reactivity in Stanford ICU patient subjects compared to HC. Statistically significant antigens were identified using false discovery rate (FDR)-adjusted p-values ($q < 0.001$), 2-fold change cutoffs, and 10,000 permutations in the Significance Analysis of Microarrays (SAM) algorithm. **(A)** 15 of 58 antigens in the cytokine array were identified with higher reactivity in ICU patients ($n = 167$) compared to HC ($n = 22$). **(B)** 10 of 55 antigens in the traditional autoantigen array had higher reactivity in ICU patients ($n = 167$) compared to HC ($n = 14$).

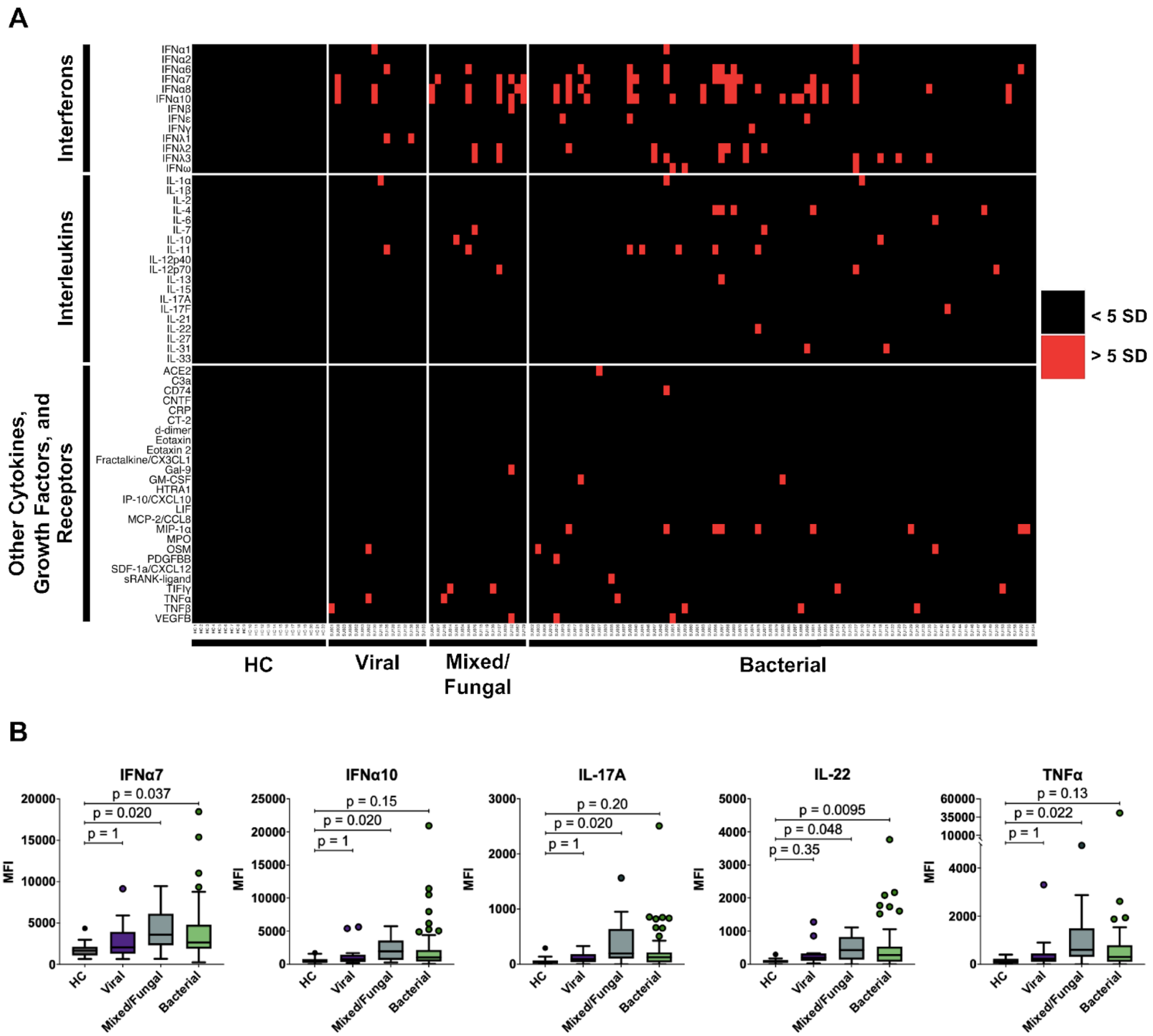

Supplemental Figure 4. Prevalence of ACA in hospitalized ICU patients with various infections. (A) Heatmap representing serum IgG ACA in the Stanford ICU patients (**Figure 1**) broken out into patients who were infected with viruses only (n = 16), a mix of pathogens (n = 16), or bacteria only (n = 83) and HC (n = 22). The Mixed/Fungal group includes 9 patients with bacterial and viral infections, 3 patients with fungal infections only, 2 patients with bacterial and fungal infections, 1 patient with viral and fungal infections, and 1 patient with bacterial, viral, and fungal infections. Cytokines are grouped on the y-axis by category (interferons, interleukins, and other cytokines/growth factors/receptors). Colors indicate ACA whose MFI measurements are > 5 SD (red) or < 5 SD (black) above the average MFI for HC. MFIs <3,000 were excluded. (**B)** Tukey box plots comparing MFI data from HC and infected Stanford ICU patients for five antigens for which statistically significant differences ($p < 0.05$) were determined between patient groups using two-tailed Wilcoxon rank-sum tests with Bonferroni correction. The middle line represents the median, while the lower and upper hinges correspond to the first and third quartiles. The upper whisker extends from the hinge to 1.5 times the interquartile range

(IQR) above the 75th percentile MFI value, and the lower whisker extends from the hinge to 1.5 times the IQR below the 25th percentile MFI value.

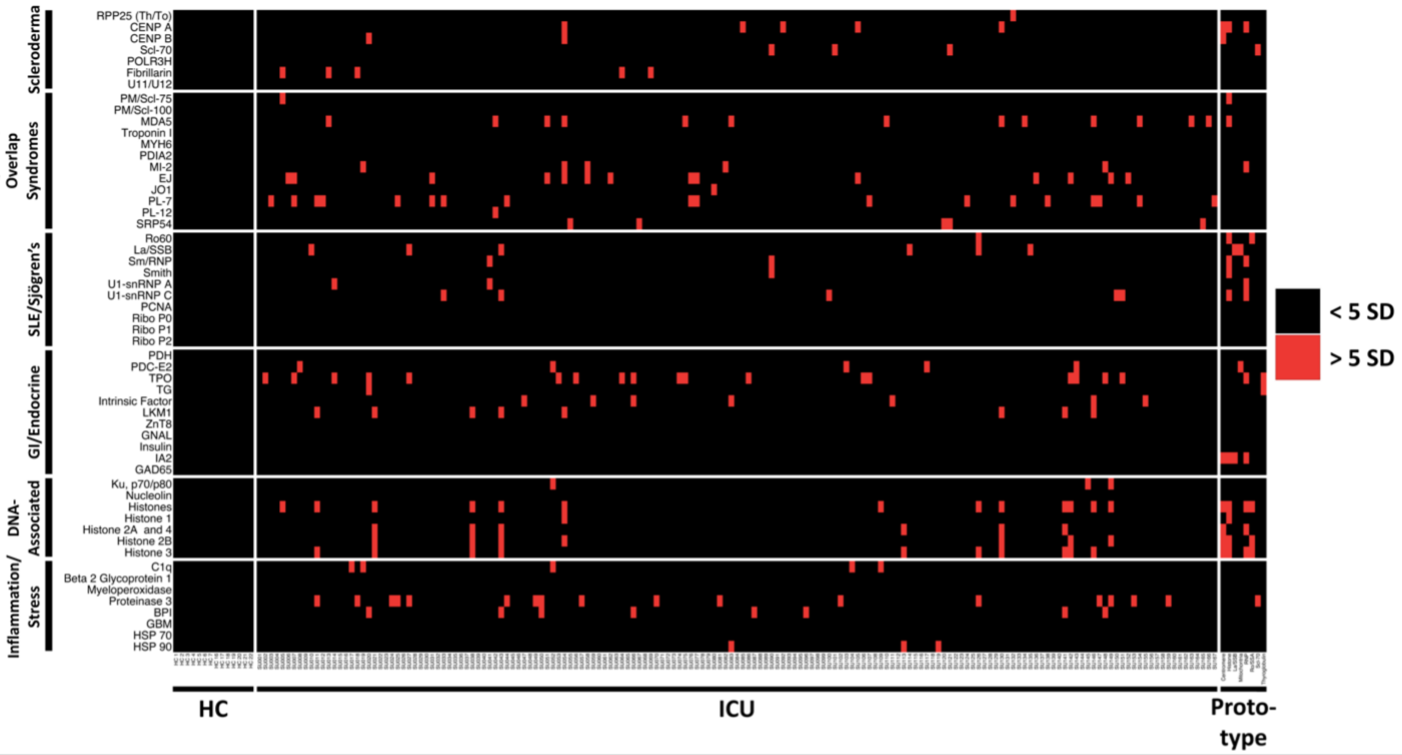

Supplemental Figure 5: Antibodies targeting traditional connective tissue disease (CTD) autoantigens in hospitalized ICU patients. Heatmap representing serum IgG antibodies measured in Stanford ICU patient subjects (n = 167), HC (n = 22), and patients with prototype autoimmune disorders (n = 8) using a 55-plex autoantigen array. Antigens are grouped on the y-axis by disease category (scleroderma, myositis and overlap syndromes such as mixed connective tissue disease (MCTD), SLE and Sjögren's, gastrointestinal and endocrine disorders); association with chromatin; and association with tissue inflammation or stress responses. Colors indicate autoantibodies whose MFI measurements are > 5 SD (red) or < 5 SD (black) above the average MFI for HC. MFIs <3,000 were excluded.

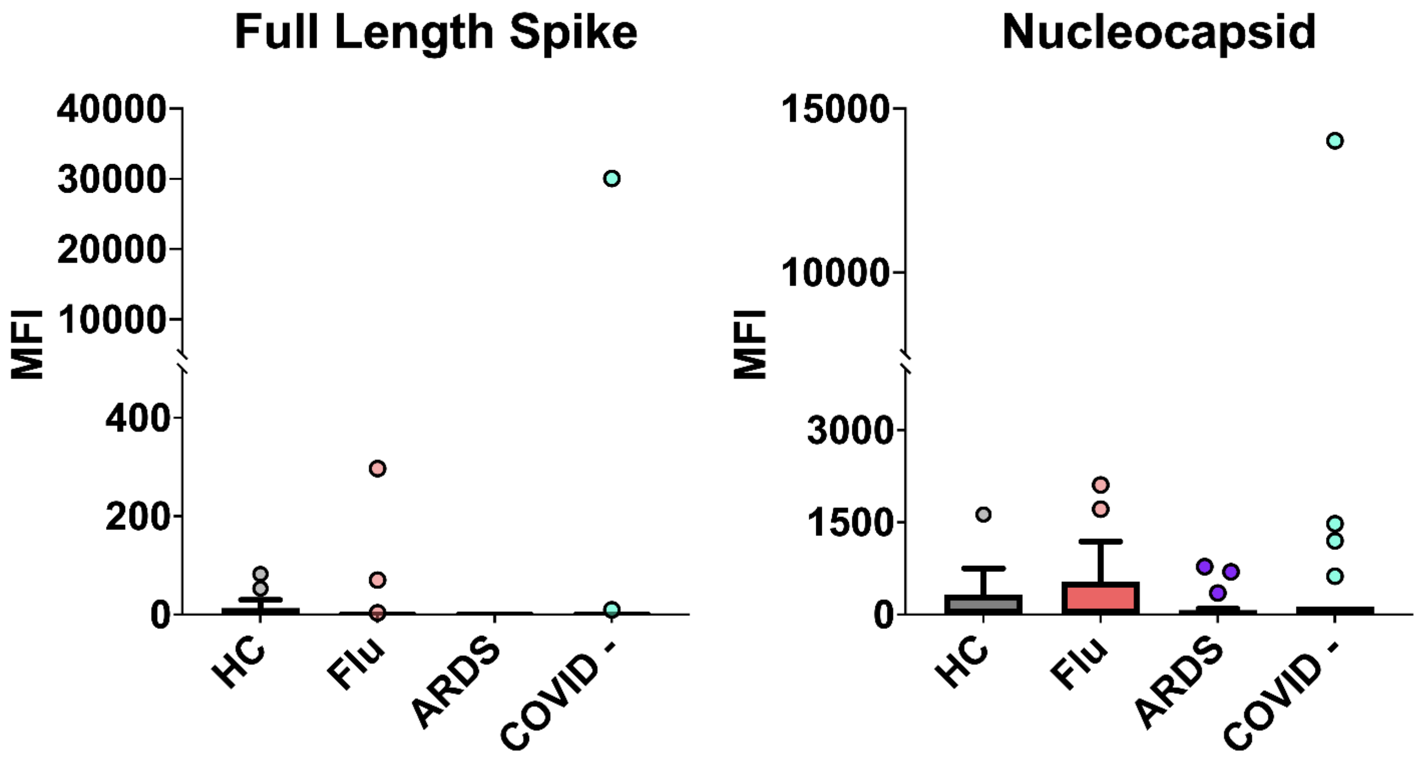

Supplemental Figure 6: A COVID-19 PCR-negative patient from the Marburg cohort has high levels of antibodies targeting SARS-CoV-2 proteins. Tukey box plots comparing MFI data for SARS-CoV-2 Spike and Nucleocapsid proteins from our viral array (2) in influenza patients (n = 25) and ARDS patients (n = 17), both collected prior to the COVID-19 pandemic; ARDS patients who were COVID-19- (n = 19); and HC (n = 11). One patient who tested negative for COVID-19 by PCR was found to have high levels of antibodies against SARS-CoV-2 spike and nucleocapsid proteins. The middle line represents the median, while the lower and upper hinges correspond to the first and third quartiles. The upper whisker extends from the hinge to 1.5 times the interquartile range (IQR) above the 75th percentile MFI value, and the lower whisker extends from the hinge to 1.5 times the IQR below the 25th percentile MFI value.

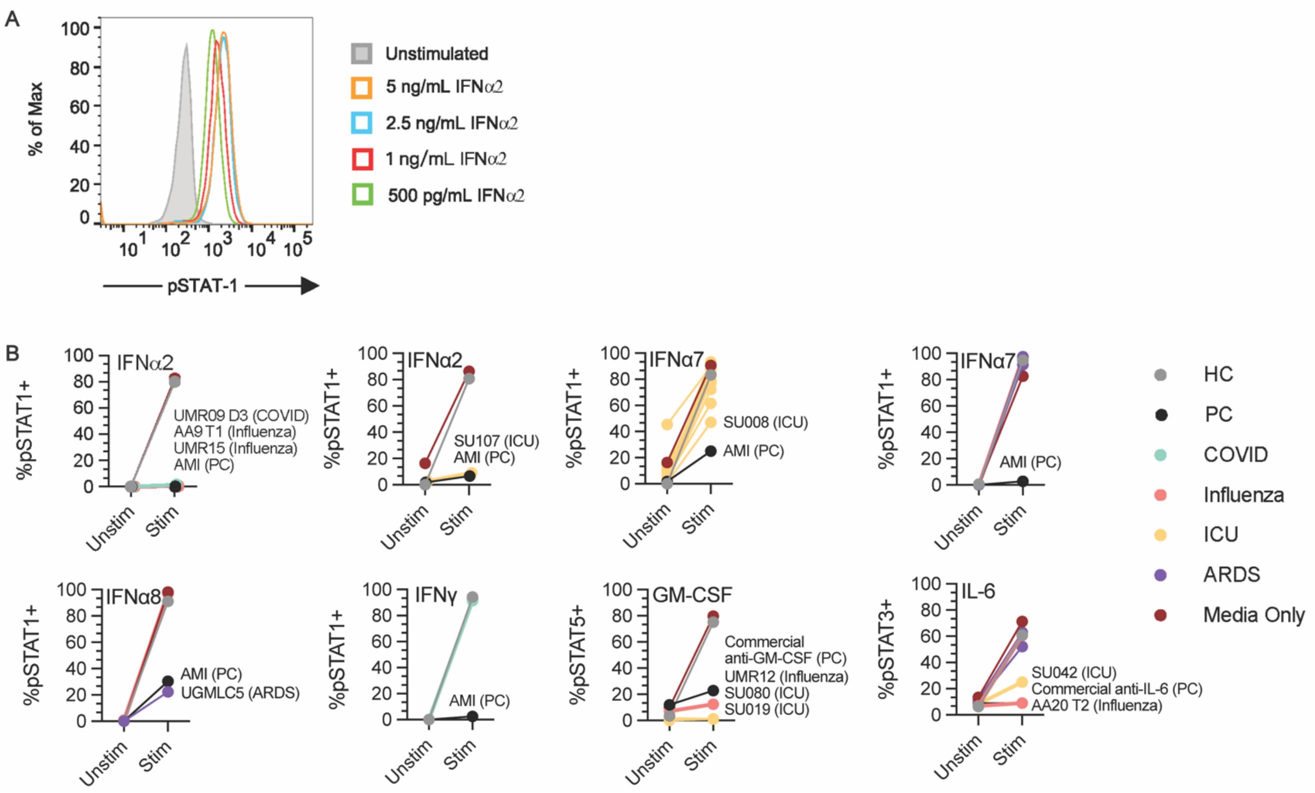

Supplemental Figure 7: Blocking Assay Development and Results. (A) Fluorescence-activated cell sorting (FACS) plots of IFNα2 blocking assay. U937 cells were treated with various concentrations of recombinant IFNα2 to identify a concentration (2.5 ng/mL) that would yield maximal stimulation in the final assay (***Supplemental Table 8***). **(B)** Blocking activity of patient serum on cells in cytokine signaling assays, reported as percentage of pSTAT positive cells in the unstimulated and stimulated condition. Patient sera were from COVID-19 (n = 2), influenza ($n_{Marburg}$ = 4, n_{Athens} = 5), Stanford ICU ($n_{infected}$ = 19, $n_{non\text{-}infected}$ = 2) and ARDS (n = 8) patients. Healthy controls (HC) and positive controls (PC: commercially available blocking antibody or prototype serum from a patient with atypical mycobacterial infection [AMI]) are also included. Independent experiments are shown. Samples with complete or partial blocking activity are labeled.

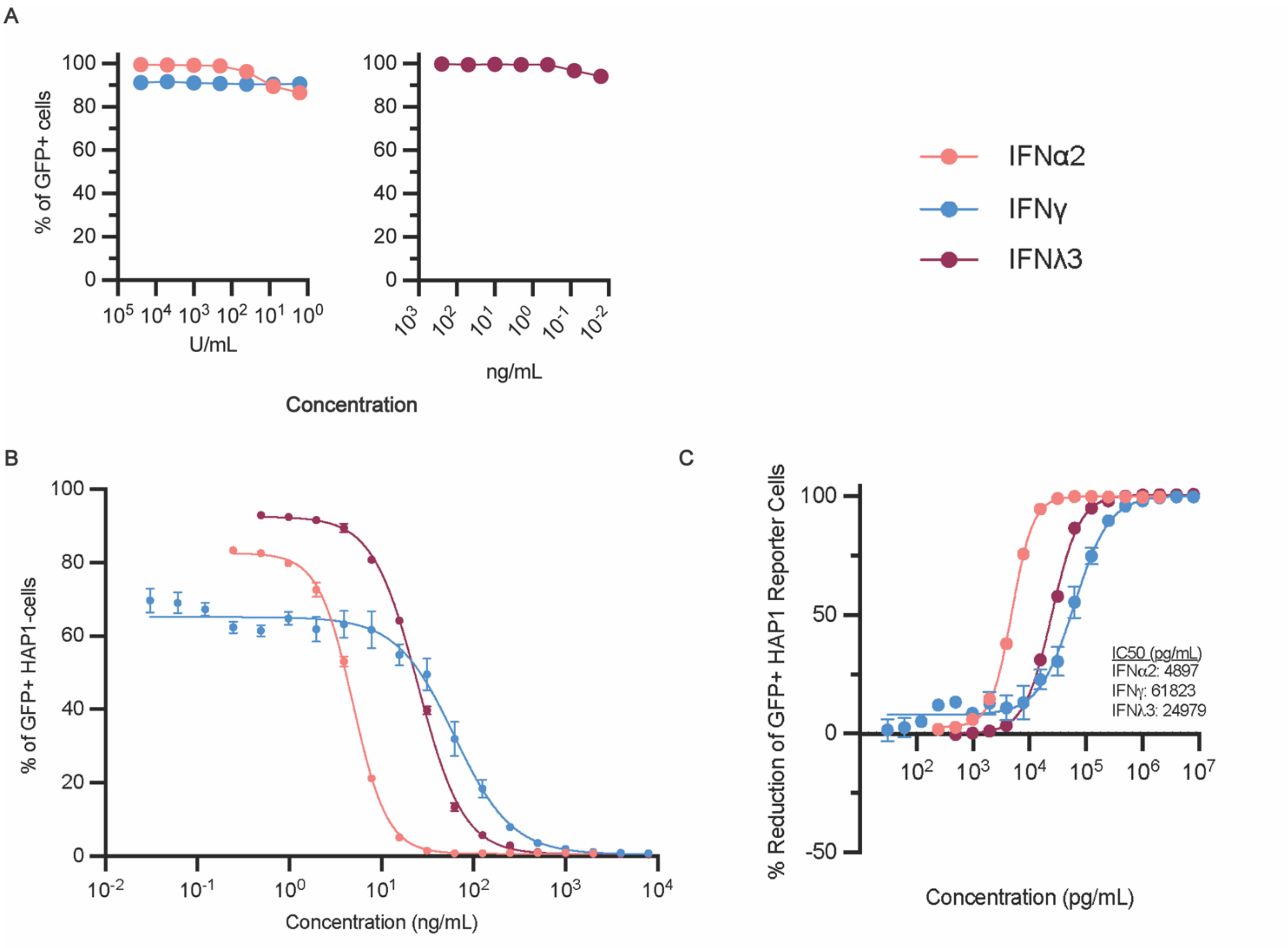

Supplemental Figure 8: HAP1 reporter cell line allows the detection of type I, II and III interferons. A human haploid reporter cell line that contains the ISRE from IFIT2 (HAP1 ISRE-GFP) was used to detect IFNs with flow cytometry 22-24 hours after incubation with IFNs. **(A)** Dose-dependent response to IFNα2, IFNγ, and IFNλ3. **(B and C)** Neutralization of IFNα2 (40 U/ml), IFNγ (8 U/ml), and IFNλ3 (1 ng/ml) with cognate monoclonal antibodies. Triplicates for each condition. Reduction of the percentages of GFP+ cells as in **(C)** was calculated by subtracting the background (no cytokines) and dividing by the percentage of GFP+ cells when the cytokines are completely functional (not incubated with neutralizing antibodies). Calculation of IC50s was done by fitting four-parameter inhibitor dose-response curves to the data, as calculated above, using GraphPad Prism v.9.3.0 (345).

SUPPLEMENTAL TABLES

Supplemental Table 1. Stanford ICU patient cohort clinical characteristics.

	ICU Patients (n = 167)
Age [Median (IQR)]	66 (54 – 75)
Sex [Percent; (N)]	
Female	42.5% (71)
Male	57.5% (96)
Self-Identified Race/Ethnicity [Percent; (N)]	
Asian	6.6% (11)
Black or African American	4.8% (8)
Native Hawaiian or Other Pacific Islander	3.6% (6)
Hispanic or Latino	19.8% (33)
White	57.5% (96)
Other/Unknown	7.7% (13)
Infection [Percent; (N)]	69.5% (116)
Median SAPS 3 [Median (IQR)]	67 (55 – 80)
Median APACHE II [Median (IQR)]	24 (19 – 32)
Shock [Percent; (N)]	58.7% (98)
30-day Mortality [Percent; (N)]	25.1% (42)

Supplemental Table 2. Marburg COVID-19 negative cohort clinical characteristics.

	COVID-19 Negative Patients (N = 19)
**Age [Median (IQR)]**	70 (57 – 81)
**Sex [Percent; (N)]**	
Female	47.4% (9)
Male	52.6% (10)
**BMI [Median (IQR)]**	24.0 (22.1 – 33.7)
**Comorbidities [Percent; (N)]**	
Diabetes	10.5% (2)
Obesity	21.1% (4)
Chronic cardiac disease	52.6% (10)
Coronary heart disease	31.6% (6)
Arterial occlusive disease	10.5% (2)
COPD	15.8% (3)
Asthma	0% (0)
Renal insufficiency	36.8% (7)
Rheumatic disorders	5.3% (1)
Neuromuscular disorders	31.6% (6)
Oncological disorders	21.1% (4)
Other immunosuppression	15.8% (3)
**Duration of hospital stay [Median; IQR]**	14.0 (8.0 – 23.0)
**Mortality [Percent; (N)]**	5.3% (1)
**Intensive Care Unit [Percent; (N)]**	52.6% (10)
Mechanical ventilation	21.1% (4)
Supplemental oxygen	63.2% (12)
**Therapy [Percent; (N)]**	
antibiotics	84.2% (16)
antivirals	21.1% (4)
antimycotics	5.3% (1)
Dialysis	10.5% (2)
Extracorporeal membrane oxygenation	0% (0)

Supplemental Table 3. Marburg Influenza patient cohort clinical characteristics.

	**Influenza Patients (n = 25)**
**Age [Median (IQR)]**	69.8 (56.5 – 81.5)
**Sex [Percent; (N)]**	
Female	52.0% (13)
Male	48.0% (12)
**BMI [Median (IQR)]**	28.2 (23.8-33.9), NA = 3
**CRP (mg/dl)**	124.3 (52.4 – 203.6)
**Hospital Mortality [Percent; (N)]**	
Yes	0% (0)
No	88.0% (22)
NA	12.0% (3)
**Ventilation [Percent; (N)]**	
Mechanical Vent (MV)	4.0% (1)
Non-Invasive Vent (NIV)	4.0% (1)
No	84.0% (21)
NA	8.0% (2)
**Days Since First Symptoms [Median (IQR)]**	6.1 (2 – 6.8), NA = 1
**Pack Years [Median (IQR)]**	22.5 (0 – 40), NA = 3

Supplemental Table 4. Giessen pneumonia-induced ARDS patient cohort clinical characteristics.

	**ARDS Patients (n = 17)**
**Age [Median (IQR)]**	56 (50 – 64.5)
**Sex [Percent; (N)]**	
**Female**	29.4% (5)
**Male**	70.6% (12)
**Secondary Diagnosis [Percent; (N)]**	
Viral pneumonia only (pathogen detected)	52.9% (9)
Bacterial pneumonia only (pathogen detected)<br>Bacterial pneumonia only (pathogen not detected)	11.8% (2)<br>17.6% (3)
Viral pneumonia with bacterial superinfection (pathogens detected)	5.9% (1)
Not Known	5.9% (1)
NA	5.9% (1)

Supplemental Table 5. Athens influenza patient cohort clinical characteristics.

	Influenza Patients (N = 40)
**Age [Median (IQR)]**	49.5 (31.5 – 66), NA = 2
**Sex [Percent; (N)]**	
Female	40.0% (16)
Male	60.0% (24)
**Hospitalizations [Percent; (N)]**	40.0% (16), NA = 3
**Hospital Mortality [Percent; (N)]**	
Yes	0% (0)
No	100.0% (40)
**Ventilation [Percent; (N)]**	
Mechanical Vent	7.5% (3)
Non-Invasive Vent	7.5% (3)
NA	85.0% (34)
**Comorbidities [Percent; (N)]**	
Diabetes	5.0% (2), NA = 3
Chronic neurological disease	2.5% (1), NA = 3
Chronic renal failure	0% (0), NA = 4
Ischemic heart disease	10.0% (4), NA = 3
COPD	20.0% (8), NA = 3
Previous Stroke	0% (0), NA = 3
Smoker	
Current	40.0% (16)
Ex	10.0% (4)
Never	37.5% (15)
E-cigarettes	2.5% (1)
NA	10.0% (4)
Alcohol consumption	10.0% (4), NA = 4
**Fever [Percent; (N)]**	92.5% (37), NA = 3
**Fever Temperature [Median (IQR)]**	38.5°C (38.2°C - 39°C), NA = 3
**Nose Symptoms [Percent; (N)]**	40.0% (16), NA = 3
**Throat Symptoms [Percent; (N)]**	37.5% (15), NA = 3
**Headache [Percent; (N)]**	45.0% (18), NA = 3
**Nausea/Vomiting [Percent; (N)]**	20.0% (8), NA = 3
**Weakness [Percent; (N)]**	82.5% (33), NA = 3
**Arthralgia [Percent; (N)]**	57.5% (23) NA = 3
**Myalgia [Percent; (N)]**	55.0% (22), NA = 3

Supplemental Table 6. Cytokine Array content.

Bead ID	Antigen	Vendor	Catalog #
1	Bare Bead		
2	Human IgG from serum	Sigma	I4506
3	Anti-Human IgG Fc fragment specific	Jackson	109-005-008
4	Anti-Human IgG (H+L)	Jackson	109-005-003
5	Anti-Human IgG F(ab') fragment specific	Jackson	109-005-006
6	CD74	Prospec	PRO-1467
7	IFNλ2	Peprotech	300-02K
10	IL-1α	Prospec	CYT-253
13	IFNα1	Prospec	CYT-291
14	IFNα7	Prospec	CYT-196
16	IFNα10	Sino Biological	10349-H08H
18	IFNα8	Sino Biological	10347-H08H
19	IFNα6	Origene	TP760329
20	IL-2	Peprotech	200-02
21	IL-4	Peprotech	200-04
22	IL-13	Peprotech	200-13
23	IL-21	Peprotech	200-21
24	Fractalkine/CX3CL1	Peprotech	300-31
25	IP-10/CXCL10	Peprotech	300-12
26	IL-31	Prospec	CYT-625
27	IL-6	Prospec	CYT-098
28	MCP-2/CCL8	Peprotech	300-15
29	OSM	Peprotech	300-10
30	IL-11	Prospec	CYT-214
31	SDF-1a/CXCL12	Peprotech	300-28A
32	IL-27	Prospec	CYT-048
33	CNTF	Prospec	CYT-272
34	CT-2	Prospec	PRO-1578
38	GM-CSF	Peprotech	300-03
39	IFNα2	R&D	11101-2
40	IFNβ	Peprotech	300-02BC
41	IFNγ	Peprotech	300-02
42	IFNε	R&D	9667-ME-025/CF
43	IFNλ1	Peprotech	300-02L
44	IFNλ3	R&D	5259-IL-025/CF
45	IFNω	R&D	11395-1
46	IL-10	Peprotech	200-10
47	IL-12p40	Peprotech	200-12P40

48	IL-12p70	Peprotech	200-12
49	IL-15	Peprotech	200-15
50	IL-17F	Peprotech	200-25
51	IL-1β	Peprotech	200-01B
52	IL-22	Peprotech	200-22
55	TNFα	Peprotech	300-01A
56	TNFβ	Peprotech	300-01B
58	ACE2	Sino Biological	10108-H05H
59	Eotaxin	Peprotech	300-21
60	Eotaxin 2	Peprotech	300-33
62	IL-17A	Peprotech	200-17
63	IL-33	Peprotech	200-33
64	IL-7	Peprotech	200-07
65	MIP-1α	Peprotech	300-08
67	PDGFBB	Peprotech	100-14B
68	sRANK-ligand	Peprotech	310-01C
69	TIFIγ	Surmodics	A11001
70	CRP	Prospec	PRO-335
71	MPO	Prospec	ENZ-074
73	C3a	R&D	3677-C3-025
76	Gal-9	R&D	9064-GA-050
77	LIF	Peprotech	300-05
78	VEGFB	Peprotech	100-20B
79	HTRA1	R&D	2916-SE-020
80	d-dimer	LeeBio	200-13-0.1

Supplemental Table 7: Number of patients with anti-cytokine antibody and connective tissue disease associated auto-antibodies by baseline characteristics and disease severity

	Variable	Total #	ACA (-)	ACA (+)	p - Value	CTD AAb (-)	CTD AAb (+)	p - Value
Baseline Characteristics	Infection							
	Uninfected	52	35	17	0.001	18	34	0.50
	Infected	115	46	69		47	68	
	Age							
	<60	56	30	26	0.41	28	28	0.04
	≥60	111	51	60		37	74	
	Sex							
	Female	71	35	36	0.88	27	44	0.87
	Male	96	46	50		38	58	
Disease Severity	Shock							
	No Shock	69	38	31	0.16	25	44	0.63
	Shock	98	43	55		40	58	
	Intubated							
	Not Intubated	67	29	38	0.34	25	42	0.75
	Intubated	100	52	48		40	60	
	30d Mort							
	Survived	125	62	63	0.72	51	74	0.47
	Died	42	19	23		14	28	

ACA (-) = No anti-cytokine antibodies
ACA (+) = Positive for anti-cytokine antibodies
CTD-AAb (-) = No connective tissue disease associated auto-antibodies
CTD-AAb (+) = Positive for connective tissue disease associated auto-antibodies
p-values calculated by Fisher's exact test

Supplemental Table 8. Traditional Autoantigen Array content.

Bead ID	Antigen	Vendor	Catalog #
1	Bare Bead		
2	Human IgG from serum	Sigma	I4506
3	Anti-Human IgG Fc fragment specific	Jackson	109-005-008
4	Anti-Human IgG (H+L)	Jackson	109-005-003
5	Anti-Human IgG F(ab') fragment specific	Jackson	109-005-006
6	Beta 2 Glycoprotein 1	Diarect	A14901
7	Myeloperoxidase	Diarect	A18501
8	La/SSB	Diarect	A12801
10	Proteinase 3	Diarect	A18601
11	Histone 1	Immunovision	HIS-1001
12	Histone 2A and 4	Immunovision	HIS-1002
13	Histone 2B	Immunovision	HIS-1003
14	CENP B	Diarect	A12501
15	Histone 3	Immunovision	HIS-1004
16	Histones	Immunovision	HIS-1000
17	GBM	Diarect	A16801
18	C1q	Biodesign	A90150H
19	BPI	Arotec	ATB01-02
24	Fibrillarin	Prospec	ENZ-566
26	IA2	Creative Biomart	PTPRN-01H
27	GAD65	Diamyd	45-08029-01
31	U11/U12	Origene	TP303746
38	CENP A	Diarect	A16901
39	EJ	Diarect	A11101
40	HSP 70	Stressgen	NSP-555
41	HSP 90	Stressgen	SPP-770
42	Intrinsic Factor	Diarect	A16701
43	JO1	Diarect	A12901
44	Ku, p70/p80	Diarect	A17301
45	LKM1	Diarect	A13501
46	MDA5	Diarect	A30001
47	MI-2	Diarect	A18101
48	PCNA	Diarect	A15401
49	PL-12	Diarect	A15701
50	PL-7	Diarect	A15601
51	PM/Scl-75	Diarect	A17001
52	Nucleolin	Diarect	A19701
53	Ribo P0	Diarect	A14101
54	Ribo P1	Diarect	A14201

55	PDC-E2	Diarect	A17901
56	Ribo P2	Diarect	A14301
57	SRP54	Diarect	A18401
58	PM/Scl-100	Diarect	A16001
59	POLR3H	Origene	TP310633
60	PDH	Sigma	P7032
62	Ro60	Diarect	A17401
65	Scl-70	Diarect	A12401
67	Sm/RNP	Immunovision	SRC-3000
68	Smith	Immunovision	SMA-3000
70	Troponin I	Prospec	PRO-1269
72	PDIA2	MyBioSource	MBS1175372
73	TG	Diarect	A12201
74	GNAL	Abnova	H00002774-P01
75	MYH6	Origene	TP313673
76	TPO	Diarect	A12101
77	ZnT8	UC Anschutz Medical Campus	
79	U1-snRNP A	Diarect	A13101
80	U1-snRNP C	Diarect	A13201
83	RPP25 (Th/To)	Origene	TP303538
84	Insulin	Sigma	I0908

Supplemental Table 9. pSTAT Induction Blocking Assay Conditions.

Cytokine	Cell Line	Concentration	Stim. Time (min)	Target	Cell Line Preparation
IFNα2	U937	2.5 ng/mL	15	pSTAT1 (Y701)	None
IFNα7	U937	1000 U/mL	15	pSTAT1 (Y701)	None
IFNα8	U937	2.5 ng/mL	15	pSTAT1 (Y701)	None
IFNγ	U937	2.5 ng/mL	15	pSTAT1 (Y701)	None
GM-CSF	U937	2.5 ng/mL	30	pSTAT5 (Y694)	None
IL-6	THP-1	1 ng/mL	20	pSTAT3 (Y705)	None

Supplemental Table 10. pSTAT Induction Blocking Assay Cytokines and Antibodies.

REAGENT	VENDOR	CATALOG #
**Cytokines**		
IFNα2	HumanKine	HZ-1066
IFNα7	PBL Assay Science	111601
IFNα8	SinoBiological	10347-H08H
IFNγ	HumanKine	HZ-1301
GM-CSF	HumanKine	GM-CSF
IL-6	HumanKine	HZ-1019
**Blocking Antibodies**		
anti-GM-CSF	R&D	AF215NA
anti-IL-6	BioLegend	501125
**Staining Antibodies**		
PE anti-STAT1 Phospho (Tyr701)	BioLegend	666404
PE anti-STAT3 Phospho (Tyr705)	BioLegend	651004
PE anti-STAT5 Phospho (Tyr694)	BioLegend	936904
PE Mouse IgG1k Isotype	BioLegend	400139
PE Rabbit IgG Isotype	Cell Signaling Technology	5742S

Supplemental Table 11. GFP Reporter Blocking Assay Cytokines and Antibodies.

REAGENT	VENDOR	CATALOG #
**Cytokines**		
IFNα2	BioLegend	592704
IFNγ	R&D Systems	285-IF-100/CF
IFNλ3	R&D Systems	5259-IL-025/CF
**Monoclonal Antibodies**		
anti-IFNα2	Invivogen	mabg-hifna-3
anti-IFNγ	Invivogen	maba-hifng-3
anti-IFNλ3	Invivogen	mabg-hil28b-3
